# DAP3 promotes mitochondrial activity and tumour progression in hepatocellular carcinoma by regulating MT-ND5 expression

**DOI:** 10.1038/s41419-024-06912-2

**Published:** 2024-07-29

**Authors:** Siyu Tan, Xiao Zhang, Xiaowei Guo, Guoqiang Pan, Lunjie Yan, Ziniu Ding, Ruizhe Li, Dongxu Wang, Yuchuan Yan, Zhaoru Dong, Tao Li

**Affiliations:** 1 Department of General Surgery, Qilu Hospital, Shandong University, Jinan, 250012 China; 2 Key Laboratory for Experimental Teratology of Ministry of Education, Key Laboratory of Infection and Immunity of Shandong Province and Department of Immunology, School of Basic Medical Science, Cheeloo College of Medicine, Shandong University, Jinan, 250012 China

**Keywords:** Cancer metabolism, Mechanisms of disease

## Abstract

Cancer cells often exhibit fragmented mitochondria and dysregulated mitochondrial dynamics, but the underlying mechanism remains elusive. Here, we found that the mitochondrial protein death-associated protein 3 (DAP3) is localized to mitochondria and promotes the progression of hepatocellular carcinoma (HCC) by regulating mitochondrial function. DAP3 can promote the proliferation, migration, and invasion of HCC cells in vitro and in vivo by increasing mitochondrial respiration, inducing the epithelial-mesenchymal transition (EMT), and slowing cellular senescence. Mechanistically, DAP3 can increase mitochondrial complex I activity in HCC cells by regulating the translation and expression of MT-ND5. The phosphorylation of DAP3 at Ser185 mediated by AKT is the key event mediating the mitochondrial localization and function of DAP3 in HCC cells. In addition, the DAP3 expression in HCC samples is inversely correlated with patient survival. Our results revealed a mechanism by which DAP3 promotes mitochondrial function and HCC progression by regulating MT-ND5 translation and expression, indicating that DAP3 may be a therapeutic target for HCC.

## Introduction

Mitochondria are dynamic organelles that supply the energy required to drive key cellular processes, such as cell survival, proliferation, and migration [1]. The mitochondrial mass is dynamically regulated by the nuclear and mitochondrial genomes (nDNA and mtDNA, respectively). Mitochondria maintain a dNTP pool to support the replication of mtDNA, which encodes at least 13 proteins, including unique ribosomal proteins for protein synthesis [2]. Mitoribosomal proteins are assembled in mitochondria, where they are required to translate mtDNA-encoded proteins [3–5]. All mtDNA-encoded proteins are synthesized within mitochondria and function in the electron transport chain (ETC) [6]. The homeostasis of the mitochondrial network is important for the intermixing of DNA and proteins among mitochondria [7], and the mitochondrial translational system responsible for synthesizing these proteins is one of the least understood intercellular systems.

As the powerhouses of eukaryotic cells, mitochondria generate ATP primarily through oxidative phosphorylation. Mitochondria are a major source of reactive oxygen species (ROS), which generally exhibit elevated levels in cancer cells and function as essential signalling molecules regulating tumour development. Mitochondrial oxidative phosphorylation has been reported to play essential roles in tumorigenesis and tumour progression [8]. Mitochondrial dysfunction leads to aberrant oxidative phosphorylation and increased resistance to glycolysis, resulting in metabolic reprogramming and lipid oxidative damage in cancers [9]. Recently, close links have been revealed between imbalanced mitochondrial dynamics and various types of cancer, including HCC. For example, decreased mitochondrial length and mitochondrial fragmentation play key roles in the proliferation and migration of HCC cells [7, 10]. Defects in mitochondria-related genes can lead to the regulation of the immune metabolic microenvironment by promoting glycolysis and lactic acid synthesis in HCC [11]. However, the molecular mechanisms by which dysregulated mitochondrial dynamics affect HCC progression remain largely unknown.

Mammalian mitochondrial ribosomes synthesize proteins that are essential for oxidative phosphorylation. Death-associated protein 3 (DAP3) localizes predominantly to mitochondria in most cells and functions as a component of the mitochondrial ribosome [12]. DAP3, also called mitochondrial ribosomal protein S29 (MRP-S29), is a GTP-binding protein located in the small subunit of the ribosome [12–14]. Immunoelectron microscopy (immuno-EM) studies have shown that DAP3 is specifically localized to the base of the lower lobe of the small subunit on the solvent side of the ribosome [15]. DAP3 has been reported to be overexpressed in many cancer types and is characterized as an oncogene [16]. However, the role and mechanisms of DAP3 in human malignancies, including HCC, are still controversial [17].

Here, we systematically analysed the dysregulated mitochondrial genes and proteins in HCC and identified DAP3 as an oncogene that promotes HCC progression by regulating the epithelial-mesenchymal transition (EMT) and cellular senescence. Molecular mechanistic studies showed that DAP3 can increase mitochondrial complex I activity by regulating the translation and expression of MT-ND5. More importantly, AKT-mediated phosphorylation at Ser185 was found to be essential for the mitochondrial localization and function of DAP3 in HCC. In addition, the level and subcellular localization of DAP3 were found to be prognostic factors, and DAP3 can serve as a potential therapeutic target for HCC.

## Methods

### Clinical specimens and patient follow-up

Samples of HCC tissues and adjacent tissues were collected from 30 HCC patients who underwent curative surgery between 2017 and 2021 at Qilu Hospital of Shandong University. Frozen tissues were subjected to mRNA or protein extraction. Another cohort of samples was based on tissue microarrays (TMAs) containing 200 pairs of HCC tissues and matched normal tissues from patients with complete clinicopathological information and follow-up data (details are listed in Table S1). This study was approved by the Institutional Ethics Committee of Qilu Hospital, Shandong University.

### Mice

Six-week-old male mice were used for all experiments. C57BL/6 mice and BALB/c nude mice were purchased from GemPharmatech (Jiangsu, China). All mice were maintained under specific pathogen-free (SPF) conditions, and the animal experimental procedures were approved by the Animal Care and Use Committee of Shandong University (Jinan, China).

### Mouse liver cancer models

Murine models of nonalcoholic fatty liver disease (NAFLD) and HCC were established in C57BL/6 mice as previously described [18]. Briefly, diethylnitrosamine (DEN; Sigma) was dissolved in phosphate-buffered saline (PBS) and injected intraperitoneally into the mice (25 mg/kg) on postnatal day 14. High-fat diet (HFD; MD12032, Medicine, Jiangsu, China) feeding by the mice commenced at 6 weeks of age.

Orthotopic HCC models were established in C57BL/6 mice as previously described [19]. Briefly, a total of 10 μg of the pCMV/SB100 and pT3-Akt/cMyc constructs mixed at a ratio of 1:25 were diluted in 2 mL of 0.9% NaCl, and the mixture was filtered and injected into the lateral tail veins of the mice within 7 to 9 s. The mice were sacrificed 6 weeks later.

Huh7 xenograft tumours were generated in male BALB/c nude mice for the tumour growth assay. Huh7 cells (2 × 10^6^) were inoculated in the inguinal region. The tumour size in each mouse was monitored every 2 days using Vernier callipers. The mice were sacrificed on day 14 after the cell injection, and the xenograft tumours were isolated and weighed.

Huh7 peritoneal hepatoma xenografts were generated in male BALB/c nude mice for the tumour metastasis assay. Huh7-GFP cells (2 × 10^6^) were injected into the abdominal cavity. After 1 week, an IVIS Spectrum imaging system (PerkinElmer) was used for tumour imaging in vivo.

### Cell culture

The human HCC cell lines Huh7, Hep3B, PLC/PRF/5, and MHCC97H are routinely maintained in our laboratory [20]. All cells were cultured in the recommended media (BasalMedia Technologies, Shanghai, China) supplemented with 10% foetal bovine serum (LONSERA; ShuangRu Biotechnology Co., Ltd., Shanghai, China) and incubated in a 37 °C incubator (YAMATO IP610, JANPN) with a humidified atmosphere containing 5% CO_2_. The cell lines were authenticated by short tandem repeat (STR) profiling and confirmed to be mycoplasma-negative.

### Transfection

The lentivirus used for DAP3 knockdown or overexpression was obtained from GeneChem (GeneChem Co., Ltd., Shanghai, China). Pools of stably transduced cells were generated by selection using puromycin (4 μg/mL) for 2 weeks. All DAP3 plasmids and small interfering RNAs (siRNAs) were purchased from Keyybio (Shandong, China). Cell transfection was performed in 6-well plates using Lipofectamine 3000 (Invitrogen, USA) according to the manufacturer’s instructions. Forty-eight hours after transfection, the cells were harvested for further experiments.

### RNA extraction and qPCR

Total RNA was extracted from human HCC tissues using TRIzol reagent (Invitrogen, Carlsbad, CA), and total RNA was extracted from HCC cells using an RNA-Quick Purification Kit (Fastagen, Shanghai, China) according to the manufacturer’s protocols. cDNA synthesis was then performed with HiScript^®^ III RT SuperMix for qPCR (+gDNA wiper) (R323; Vazyme Biotech Co., Ltd.). RNA expression was measured using ChamQ Universal SYBR qPCR Master Mix (Q311; Vazyme Biotech Co., Ltd., Nanjing, China) and a Bio-Rad CFX Connect^™^ Real-Time system (USA), and relative RNA abundances were calculated using the 2^ΔΔCt^ method with normalization to actin. All reactions were conducted in triplicate, and three independent experiments were performed. All primers were obtained from Tsingke Biotechnology Co., Ltd. (Beijing, China), and a summary of the primers used is provided in Table S2.

### Western blotting

Total protein was extracted from tissues or cells via lysis in precooled radioimmunoprecipitation assay (RIPA) buffer (Solarbio, Beijing, China) supplemented with protease and phosphatase inhibitors (Solarbio, Beijing, China). An Enhanced BCA Protein Assay Kit (Beyotime, Shanghai, China) was used to determine the protein concentration in each supernatant. Samples containing equal amounts of protein were subjected to separation by sodium dodecyl sulfate-polyacrylamide gel electrophoresis (SDS‒PAGE) and electrophoretically transferred onto 0.2 μm polyvinylidene fluoride (PVDF) membranes (Millipore, USA). After being blocked with 5% nonfat milk, the membranes were incubated with the corresponding primary antibodies at 4 °C overnight. After washes with TBST, the membranes were incubated with suitable horseradish peroxidase (HRP)-conjugated secondary antibodies (Zhongshan Golden Bridge Biological Technology Co., Ltd., Beijing, China). The immunocomplexes were visualized using an enhanced chemiluminescence kit (Vazyme, Nanjing, China), and images were acquired using Tanon-4800 software. All the antibodies used in this study are listed in the Additional file: Table S2.

### Immunohistochemical (IHC) staining

IHC staining was performed using a two-step mouse/rabbit-enhanced polymer detection system (Zhongshan Golden Bridge Biological Technology Co., Ltd., Beijing, China) according to the manufacturer’s instructions. Briefly, after deparaffinization in xylene and rehydration through a graded ethanol series, the samples were subjected to ethylenediaminetetraacetic acid (EDTA)-mediated high-temperature antigen retrieval. Following the blockade of endogenous peroxidase activity by incubation with 3% hydrogen peroxide, the tissue sections were incubated with a primary antibody overnight at 4 °C. After sequential incubations with reactive polyethyleneimine and enhanced enzyme-labelled goat anti-mouse/rabbit IgG polymer, the colour was developed with a 3,3’-diaminobenzidine (DAB) solution (Zhongshan Golden Bridge Biological Technology Co., Ltd., Beijing, China) under a microscope, and the samples were counterstained with hematoxylin. DAP3 expression was scored according to both the percentage and intensity of positively stained cells: 0–5%, 0; 6–35%, 1; 36–70%, 2; and more than 70%, 3. The final scores were considered to indicate low or high expression as follows: low expression (score of 0–1) and high expression (score of 2–3). The scores were determined independently by two experienced pathologists in a blinded manner, and the mean percentages were calculated.

### Cell viability and proliferation assays

Cell viability was assessed at the indicated time points using a Cell Counting Kit (Elabscience, Wuhan, China) according to the manufacturer’s instructions. Briefly, transfected cells were seeded into 96-well plates at a density of 2 × 10^3^ cells per well, with six replicate wells. The cells in each well were incubated with 10 µL of Cell Counting Kit-8 (CCK-8) solution at 37 °C for 1 hour in the dark. The absorbance at 450 nm was measured using a multifunctional microplate reader (TECAN M200) on 5 consecutive days.

### Migration and invasion assays

Cell migration and invasion assays were performed using 24-well plates containing 8-μm pore size Transwell filter inserts (BIOFIL, Guangzhou, China) precoated with or without diluted Matrigel (Corning, NY, USA). HCC cells (5 × 10^4^) in serum-free medium were seeded into the upper chambers, and medium supplemented with 20% foetal bovine serum (FBS) was subsequently added to the bottom chambers. After an incubation at 37 °C for 48 h, the cells remaining on the upper surface of each insert were gently removed by wiping with cotton swabs. The cells remaining on the Transwell chamber membranes were fixed with 4% paraformaldehyde (PFA) and then stained with 0.1% crystal violet. The cells on the underside of each membrane were counted using an inverted fluorescence microscope (cells were counted in at least three randomly selected fields).

### Transmission electron microscopy (TEM)

The cells were fixed overnight at 4 °C with 2.5% glutaraldehyde, incubated with 1% osmium tetroxide for 1 h at 4 °C, dehydrated through a graded ethanol series, saturated through a graded acetone series, sliced into 50-nm ultrathin sections, stained with 3% uranium acetate/lead citrate and viewed using a JEM-1200 EX II electron microscope (JEOL, Japan).

### Seahorse assays

For the extracellular assay, 2 × 10^5^ Huh7 cells were seeded in a Seahorse Bioscience culture plate in XF Base Medium Minimal DMEM (Agilent, California, USA) supplemented with 10 mM glucose and 2 mM glutamine in a non-CO_2_ incubator for 1 h. The basal oxygen consumption rate (OCR) was measured over a 30 min period. The cells were treated with 2 mM oligomycin, 1.5 mM FCCP, and rotenone and antimycin A (1 mM each) (all drugs were obtained from Agilent Technologies). The basal OCR, maximum respiration, and ATP production were measured with an XF96 Seahorse Extracellular Flux Analyzer (Agilent, California, USA) according to the manufacturer’s instructions.

### Measurements of mitochondrial mass, mitochondrial membrane potential, and mitochondrial ROS levels

Huh7 cells were stained with 100 nM MitoTracker Green (Invitrogen, NY, USA) in PBS for 30 min at 37 °C and then subjected to a flow cytometry analysis (Gallios, Beckman Coulter) to measure the mitochondrial mass. Huh7 cells were stained with 100 nM MitoTracker Red CMXRos (Invitrogen, NY, USA) in PBS for 30 min at 37 °C to measure the mitochondrial membrane potential. After two washes with PBS, the cells were analysed using flow cytometry (Gallios, Beckman Coulter). Cells were treated with 10 μM MitoSOX (Invitrogen, NY, USA) to measure mitochondrial ROS levels. After two washes with PBS, the cells were analysed using flow cytometry (Gallios, Beckman Coulter).

### Immunofluorescence staining

For mitochondrial imaging, Huh7 cells were stained with MitoTracker Red CMXRos for 30 min and then fixed with 4% PFA for nuclear staining with 4’,6-diamidino-2-phenylindole (DAPI). Slides were viewed using an Olympus DP80 microscope (Olympus, Japan).

### β-Galactosidase (β-Gal) staining

Senescence-associated β-galactosidase (SA-β-Gal) staining was performed using a kit (Thermo Fisher Scientific, MA, USA) according to the manufacturer’s protocol.

### Determination of the NAD^+^/NADH ratio

The NAD^+^/NADH ratio was determined using an NAD^+^/NADH ratio determination kit (Abcam, Massachusetts, USA) according to the manufacturer’s protocol.

### Mitochondrial complex I enzyme activity measurement

Mitochondrial complex I enzyme activity was measured using a complex I enzyme activity microplate assay kit (Abcam, Massachusetts, USA) according to the manufacturer’s protocol.

### ATP measurement

ATP production was measured using an ATP determination kit (Sigma-Aldrich, St. Louis, USA) according to the manufacturer’s protocol.

### RNA sequencing (RNA-seq) analysis

The sequencing data were filtered with SOAPnuke (v1.5.2) by (1) removing reads containing sequencing adaptors, (2) removing reads with a low-quality base (base quality ≤ 5) ratio >20%, and (3) removing reads with an unknown base (“N” base) ratio >5%. Clean reads were obtained and stored in FASTQ format. The raw data were uploaded to the Sequence Read Archive (accession no. PRJNA941106). The clean reads were mapped to the reference genome using HISAT2 (v2.0.4). Bowtie2 (v.2.2.5) was applied to align the clean reads to the reference coding gene set, and the gene expression levels were then calculated with RSEM (v1.2.12). Essentially, the differential expression analysis was performed using the Poisson algorithm with threshold criteria of a false discovery rate (FDR) ≤ 0.001 and |Log2 ratio| ≥1.

Then, Kyoto Encyclopedia of Genes and Genomes (KEGG; https://www.kegg.jp/) enrichment analysis of the annotated differentially expressed genes (DEGs) was performed with phyper (https://stat.ethz.ch/R-manual/R-devel/library/stats/html/Hypergeometric.html) based on a hypergeometric test to obtain insights into the change in phenotype. The *p* values for terms and pathways were adjusted by calculating the q values with a rigorous threshold (q ≤ 0.05) or by the Bonferroni correction.

For the gene set variation analysis (GSVA), we calculated the enrichment score for each sample in the TCGA-LIHC gene set using the R package GSVA (v1.40.1) and predefined the gene ranking. The gene set h.all.v7.4.symbols.gmt was downloaded to evaluate related pathways and molecular mechanisms, and the enrichment score matrix was obtained.

For the gene set enrichment analysis (GSEA), we used GSEA (http://software.broadinstitute.org/gsea/index.jsp) software (v3.0) and divided the samples into two groups according to DAP3 expression. The Molecular Signatures Database (http://www.gsea-msigdb.org/gsea/downloads.jsp) was used to assess pathways and molecular mechanisms. A *P* value < 0.05 and an FDR < 0.25 were considered to indicate statistically significant enrichment.

### Statistical analysis

Statistical significance was determined using GraphPad Prism (v8.3.0) software. Two-tailed unpaired or paired Student’s t tests and two-way ANOVA were used to determine the significance of differences between two groups and among more than two groups, respectively. The difference in overall survival (OS) was tested using the log-rank test. The data are presented as the means±standard deviations (SDs).

## Results

### Elevated DAP3 expression is correlated with shorter survival of HCC patients

**Fig. 1:**
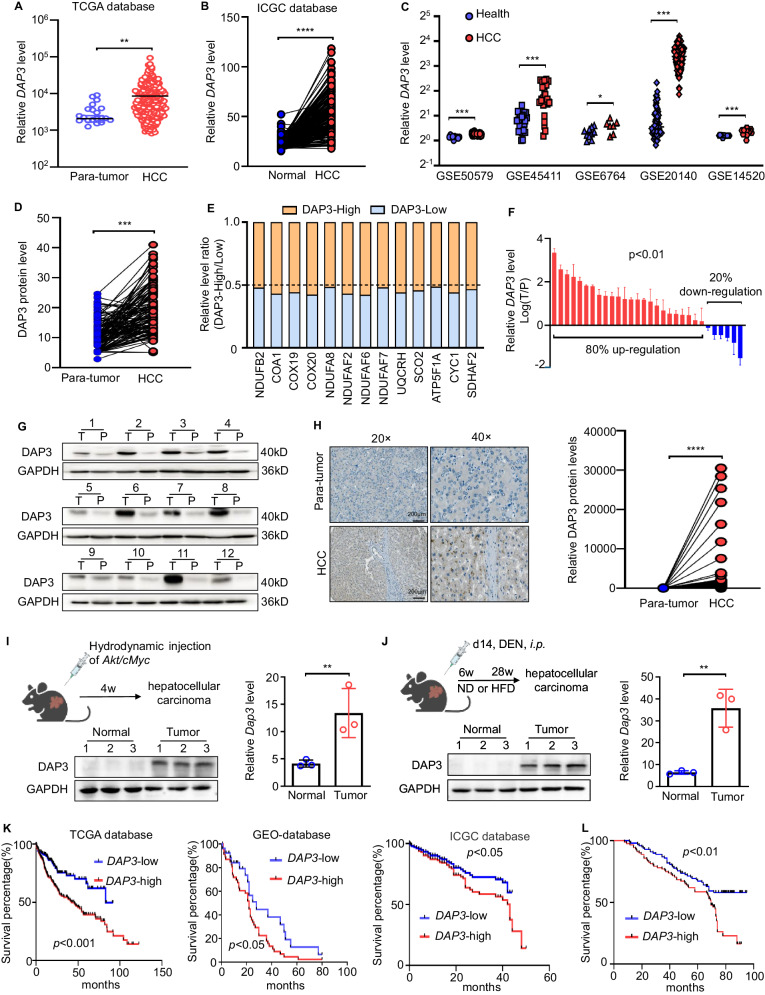
Elevated DAP3 expression is correlated with shorter survival of HCC patients. **A** The expression of the DAP3 mRNA in HCC and para-tumour liver samples was evaluated by analysing RNA-seq data from TCGA database. **B** Differential expression profiles of DAP3 in HCC and matched normal liver tissues from the ICGC cohort. **C** The DAP3 mRNA level in HCC samples was evaluated in several GEO datasets. **D** DAP3 protein expression in HCC and matched para-tumour tissues from the CPTAC database. **E** Analysis of the relationship of mitochondrial gene (*NDUFB2, COA1, COX19, COX20, NDUFA8, NDUFAF2, NDUFAF6, NDUFAF7, UQCRH, SCO2, ATP5F1A, CYC1* and *SDHAF2*) expression with *DAP3*. Data were obtained from the TCGA-LIHC database. **F** qPCR was performed to measure the DAP3 mRNA level in 30 paired HCC tissues and para-tumour liver tissues. **G** Western blotting was used to measure the DAP3 protein level in HCC tissues and paired para-tumour liver tissues (*n* = 12). **H** Representative IHC images of DAP3 staining in human HCC tumour and para-tumour liver tissues. (Scale bar: 200 μm; magnification: 20× and 40×). Dot plots showed the comparison between para-tumour liver tissues and tumour tissues. The positive rate was measured using ImageJ software. ****p < 0.0001. **I** Orthotopic HCC models were established by a hydrodynamic injection of plasmids containing the Akt and cMyc genes with the SB100 transposase into C57BL/6 mice. The relative mRNA and protein expression levels of DAP3 were measured using qPCR and western blot analysis, respectively (representative of three independent experiments). **J** An HFD-induced model of HCC was induced by subcutaneously injecting 25 mg/kg DEN into mice on postnatal day 14, and HFD feeding commenced at 6 weeks of age. The relative mRNA and protein expression levels of DAP3 were measured using qPCR and western blot analysis, respectively (representative of three independent experiments). **K** Kaplan‒Meier analysis of the correlations between DAP3 expression and the survival rate of HCC patients based on an analysis of TCGA, GEO, and ICGC databases. **L** Kaplan‒Meier survival curves for HCC patients with high (*n* = 100) or low (*n* = 100) DAP3 expression. The cut-offs used for grouping were determined based on the median IHC score. Statistical analysis was performed using 2-tailed Student’s t test (Fig. 1A, B, C, D, F, H, I, and J) or the log-rank (Mantel‒Cox) test (Fig. 1K, L). The error bars indicate the means ± SDs. n.s., not significant; *p < 0.05; **p < 0.01; ***p < 0.001; and ****p < 0.0001.

We systematically analysed several databases of mitochondrial or total gene and protein expression data to identify the dysregulated mitochondrial proteins in HCC (Supplementary Fig. S1A). First, the MitoCarta 3.0 inventory [21] and the Gene Ontology (GO) term mitochondrion [GO:0005739] database were analysed, and 722 genes regulating mitochondrial dynamics were obtained (Supplementary Fig. S1B). Then, data from human HCC patients in The Cancer Genome Atlas (TCGA) cohort, The International Cancer Genome Consortium (ICGC) cohort (with control data from normal tissues obtained from the Genotype–Tissue Expression [GTEx] database), the Gene Expression Omnibus (GEO) database (GSE14520) [22] and a proteomics dataset [23] were further examined. From these databases, DAP3 was selected as an upregulated gene and identified as a potential target regulating mitochondrial function in HCC (Fig. 1A, B, Supplementary Fig. S1C). Indeed, several public GEO HCC datasets, such as GSE50579, GSE45411, GSE6764, and GSE20140, and the Clinical Proteomic Tumor Analysis Consortium (CPTAC) database [24] also showed that DAP3 expression was higher in HCC tissues than in normal liver tissues or matched para-tumour samples (Fig. 1C, D). More importantly, the elevated DAP3 mRNA level in HCC tissues was positively correlated with increased expression of genes related to mitochondrial function, mitochondrial respiration, ETC (Fig. 1E, Supplementary Fig. S1D).

Then, we examined DAP3 expression in HCC and corresponding para-tumour tissues collected from both clinical patients and orthotopic HCC mouse models to further confirm the high DAP3 level in HCC in the analyses of those profiles. These results also showed that DAP3 expression in HCC tissue samples was higher than that in the corresponding para-tumour samples (Fig. 1F–J).

Then, we evaluated the correlation between the DAP3 level in HCC samples and the patient prognosis. First, patients with high DAP3 expression in HCC tissues had significantly shorter OS than those with low DAP3 expression according to TCGA, GEO, and ICGC databases (Fig. 1K). A survival analysis based on an IHC analysis of the TMA also indicated that HCC patients with higher DAP3 expression had markedly shorter OS (Fig. 1L). These data indicated that DAP3 is significantly upregulated in HCC samples and that the elevated DAP3 level in HCC tissues is correlated with a poor patient prognosis.

### DAP3 promotes the proliferation, migration, and invasion of HCC cells in vitro and in vivo

**Fig. 2:**
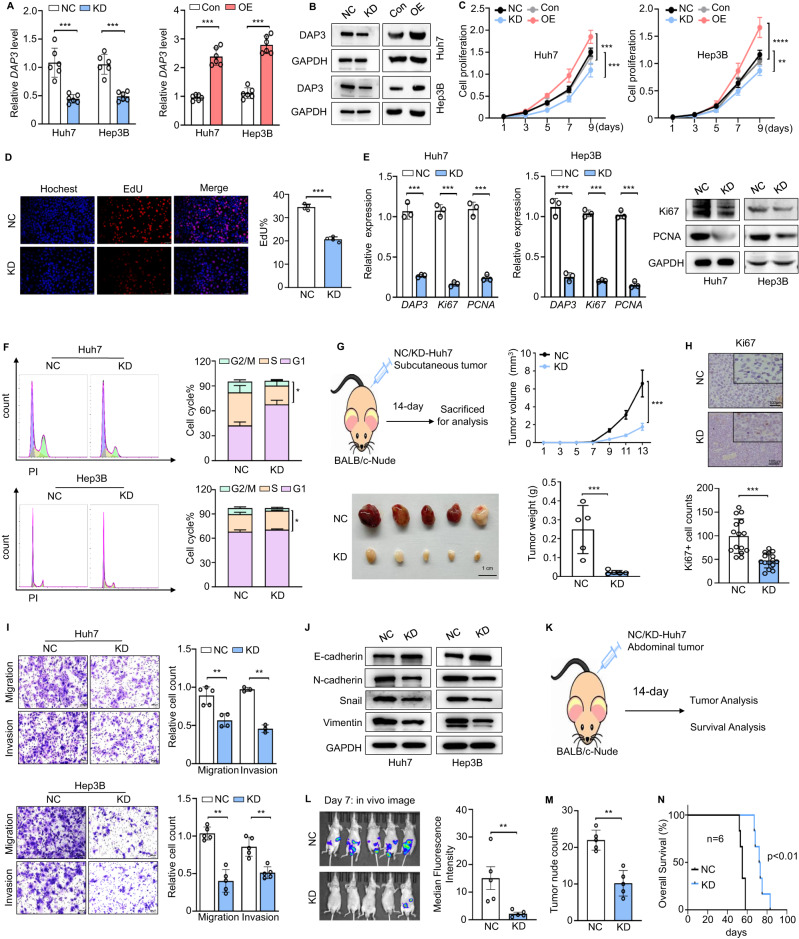
DAP3 promotes the proliferation, migration, and invasion of HCC cells. **A**, **B** The efficiency of DAP3 knockdown (KD) and overexpression (OE) in Huh7 and Hep3B cells was verified using qPCR and western blotting (representative of three independent experiments). **C** CCK-8 assays were used to evaluate the proliferative capacity of two HCC cell lines with DAP3 knockdown or overexpression (representative of three independent experiments). **D** EdU incorporation assays (EdU, red; Hochest, blue) were conducted in Huh7 cells to compare the percentage of S phase cells (representative of three independent experiments). Scale bars, 50 μm. Hoechst staining was used to indicate total cells, while EdU incorporation indicated cells with active DNA replication. **E** The effect of DAP3 on the expression of proliferation-related genes and proteins (Ki67 and PCNA) in the Huh7 and Hep3B cell lines was verified using qPCR and western blot analysis (representative of three independent experiments). **F** The cell cycle distribution of Huh7 and Hep3B cells after DAP3 knockdown was determined using flow cytometry (representative of three independent experiments). **G** Subcutaneous tumour models were established with stable DAP3-KD Huh7 cells and NC Huh7 cells. Scale bar, 1 cm. Tumour volumes were recorded consecutively to generate tumour growth curves. Then, the tumours were collected from the sacrificed mice and weighed. **H** Immunohistochemical staining was performed to visualize the expression of Ki67 in tumour samples. Scale bar, 100 μm; magnification, 20× and 40×. The bar charts show the relative Ki67^+^ cell counts. **I** Transwell assays were used to measure the migration and invasion of Huh7 and Hep3B cells. Scale bars, 200 μm. The bar charts show the relative counts (relative to the NC group) of cells that passed through the chamber membrane (representative of three independent experiments). **J** Expression of EMT-related proteins (E-cadherin, N-cadherin, Snail, and Vimentin) in Huh7 and Hep3B cells with DAP3 knockdown. **K**–**N** Mouse models of metastasis were established by i.p. injecting Huh7-GFP cells into BALB/c nude mice. The experimental design (**K**), tumour images and quantification of the tumour burden on day 7 (**L**), the tumour nodule count on day 14 (**M**), and survival curves (**N**) are shown. Statistical analysis was performed using 2-tailed Student’s t test (Fig. 2A, D, E, F, G, H, I, L, and M), two-way ANOVA (Fig. 2C, G), or the log-rank (Mantel‒Cox) test (Fig. 2N). The error bars indicate the means ± SDs. n.s., not significant; *p < 0.05; **p < 0.01; ***p < 0.001; and ****p < 0.0001. KD knockdown, NC negative control, OE overexpression, Con control.

As an approach to explore the function of DAP3 in HCC progression, we selected Huh7 and Hep3B cells to establish cell lines with DAP3 knockdown (KD) or overexpression (OE) based on their basal expression levels (Fig. 2A, B; Supplementary Fig. S2A, B). CCK-8 and EdU incorporation assays showed that DAP3-KD significantly inhibited cell proliferation and decreased the expression of Ki67 and PCNA in Huh7 and Hep3B cells, while DAP3-OE significantly promoted the proliferation of HCC cells (Fig. 2C–E, Supplementary Fig. S2C). Flow cytometry analysis revealed that DAP3-KD significantly increased the percentage of G0/G1-phase cells but decreased the percentage of G2/M-phase cells (Fig. 2F), whereas DAP3-OE had the opposite effect (Supplementary Fig. S2D). Finally, HCC cells were injected subcutaneously into the right groins of nude mice, and the DAP3-KD cells showed a significantly decreased ability to form tumours, accompanied by reduced Ki67 expression in nude mice (Fig. 2G, H). These results indicate that DAP3 promotes HCC cell proliferation and tumour growth both in vitro and in vivo.

Transwell migration and Matrigel invasion assays showed that DAP3-KD significantly inhibited the migration and invasion of HCC cells (Fig. 2I). Intriguingly, the morphology of the DAP3-KD cells changed from an elongated/irregular shape to an epithelial cobblestone shape (Supplementary Fig. S2E). We further investigated whether DAP3 affects the EMT process in HCC cells. As expected, DAP3-KD downregulated N-cadherin, Vimentin, and Snail expression and upregulated E-cadherin expression, and the opposite effects were observed when DAP3 was overexpressed (Fig. 2J, Supplementary Fig. S2F). Then, BALB/c nude mice were subjected to an intraperitoneal (i.p.) injection of Huh7-GFP cells (2 × 10^6^) with or without stable DAP3-KD to establish the HCC model in vivo (Fig. 2K). The chemiluminescence assay showed that mice injected with DAP3-KD Huh7 cells developed fewer metastases in the abdominal cavity and fewer tumour nodules in the intestine (Fig. 2L, M). The OS time was also significantly longer in the DAP3-KD group (Fig. 2N). These results suggest that DAP3 can promote HCC cell invasion and metastasis by regulating the EMT process.

### DAP3 improves mitochondrial function and reprograms metabolism in HCC

**Fig. 3:**
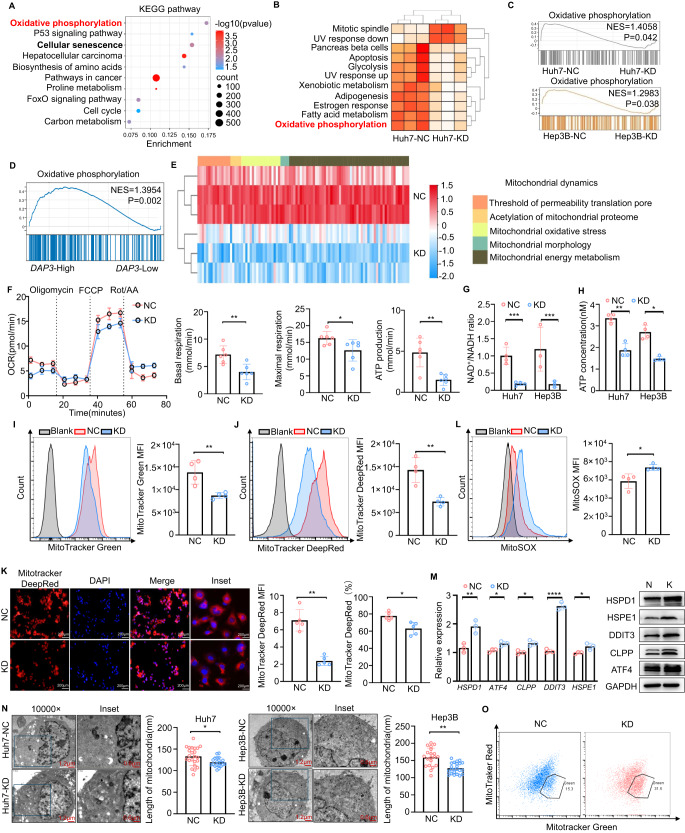
DAP3 improves mitochondrial function and reprograms metabolism in HCC cells. **A**–**C** Comparison of RNA-seq data between DAP3-KD and NC HCC cells. **A** A KEGG pathway enrichment analysis of the differentially expressed genes in Huh7 cells was performed. The top 10 significantly enriched KEGG pathways (*p* < 0.05) are shown. **B** Heatmap indicating the enrichment of specific hallmark pathways in DAP3-KD cells compared with Huh7-NC cells determined using GSVA. **C** Results of GSEA comparing the enrichment of the oxidative phosphorylation pathway between DAP3-KD and NC HCC cells. **D** GSEA of TCGA-LIHC cohort revealed a positive correlation between oxidative phosphorylation and DAP3 expression. **E** Heatmap showing the analysis of the expression of proteins related to mitochondrial dynamics in the DAP3**-**NC/KD Huh7 cells. **F** Representative OCR measurements in DAP3-NC/KD Huh7 cells after the addition of oligomycin, the mitochondrial decoupler FCCP, or rotenone + antimycin. Basal and maximal respiration and ATP production were quantitatively calculated. **G** The NAD^+^/NADH ratio was determined by performing a colorimetric WST-8 assay (representative of three independent experiments). **H** ATP production was determined using an ATP Bioluminescent Assay Kit (representative of three independent experiments). **I**, **J** Flow cytometry analysis of the mitochondrial mass and mitochondrial membrane potential in DAP3-NC/KD Huh7 cells labelled with MitoTracker Green (**I**) and MitoTracker DeepRed (**J**) (representative of three independent experiments). **K** Fluorescence staining (MitoTracker DeepRed, red; DAPI, blue) showing the mitochondrial morphology and mitochondrial membrane potential in the DAP3-NC/KD Huh7 cells. Scale bar, 200 μm. The MFI of MitoTracker Red and the number of MitoTracker Red+ cells were determined using ImageJ software. **L** Representative FACS plots and mean fluorescence intensity (MFI) of MitoSOX in DAP3-NC/KD Huh7 cells (representative of three independent experiments). **M** qPCR and western blot analyses of the expression of UPR_mt_-related genes and proteins (HSPD1, ATF4, CLPP, DDIT3, and HSPE1) in DAP3-NC/KD Huh7 cells (representative of three independent experiments). **N** TEM images and statistical graphs showing the mitochondrial morphology in the DAP3-NC/KD Huh7 and Hep3B cells. The mitochondrial length was calculated using ImageJ software. Scale bars, 1.2 μm (10,000×) and 0.6 μm (inset). **O** FACS analysis of the mitochondrial membrane potential in DAP3-NC/KD Huh7 cells (representative of three independent experiments). Statistical analysis was performed using a 2-tailed Student’s t test (Fig. 3F, G, H, I, J, K, L, M, and N). The error bars indicate the means ± SDs. n.s., not significant; *p < 0.05; **p < 0.01; ***p < 0.001; and ****p < 0.0001.

We further performed RNA-seq and proteomic analyses of DAP3-KD/negative control (NC) Huh7 and Hep3B cells to understand the mechanism underlying DAP3-mediated HCC progression. The volcano plot generated from the RNA-seq data showed 1352 DEGs (867 upregulated and 485 downregulated genes) in the DAP3-KD Huh7 cells and 1131 DEGs (577 upregulated and 554 downregulated genes) in the DAP3-KD Hep3B cells compared to the corresponding NC cells (Supplementary Fig. S3A). Both the KEGG pathway enrichment analysis and GSVA revealed that oxidative phosphorylation was one of the most significantly enriched pathways in the DAP3-KD group compared with the NC group (Fig. 3A, B). The EMT signalling pathway was also significantly enriched in the DAP3-NC group (Supplementary Fig. S3B).

GSEA revealed the positive enrichment of gene sets encoding oxidative phosphorylation (the Broad Hallmark Gene Set)- and ATPase activity (Gene Ontology Term)-related molecules in the DAP3-NC groups of both Huh7 and Hep3B cells (Fig. 3C, Supplementary Fig. S3C), revealing that DAP3 plays an important role in mitochondrial activity in HCC cells. Similarly, an analysis of the TCGA database revealed a positive relationship between oxidative phosphorylation activity and DAP3 expression (Fig. 3D). A heatmap of the proteomic data also showed that mitochondrial activity in DAP3-NC cells was greater than that in DAP3-KD cells (Fig. 3E). These data suggest that DAP3 may regulate mitochondrial function in HCC cells.

We further attempted to determine the effect of DAP3 on mitochondrial function and found that DAP3-KD could reduce basal and maximal respiration, as well as ATP production, but had no obvious effects on glycolysis or the glycolytic capacity (Fig. 3F, Supplementary Fig. S3D). Consistently, reduced cellular NAD^+^ and ATP levels, as well as a reduced mitochondrial mass and mitochondrial membrane potential, were detected in DAP3-KD HCC cells (Fig. 3G–K), while the cellular ROS level was significantly increased upon mitochondrial fusion (Fig. 3L). In addition, DAP3 knockdown activated the mitochondrial unfolded protein response (UPRmt), as indicated by the increased expression of several genes, such as DDIT3, to combat mitochondrial dysfunction (Fig. 3M).

Finally, the effect of DAP3-KD on mitochondrial morphology was further explored. TEM revealed that the DAP3-KD HCC cells had fragmented mitochondria, swollen cristae, and shorter mitochondrial lengths, and these changes could be reversed by DAP3 reintroduction (Fig. 3N, Supplementary Fig. S3E, F). Moreover, DAP3-KD caused the accumulation of mitochondria in Huh7 cells with a MitoTracker Green^high^ and MitoTracker DeepRed^low^ phenotype (Fig. 3O, Supplementary Fig. S3G), indicating a subset of cells with dysfunctional mitochondria [25, 26]. Taken together, these results indicate that DAP3 is important for proper mitochondrial morphology and function in HCC cells.

### DAP3 modulates mitochondrial function and HCC progression by increasing mitochondrial complex I activity

**Fig. 4:**
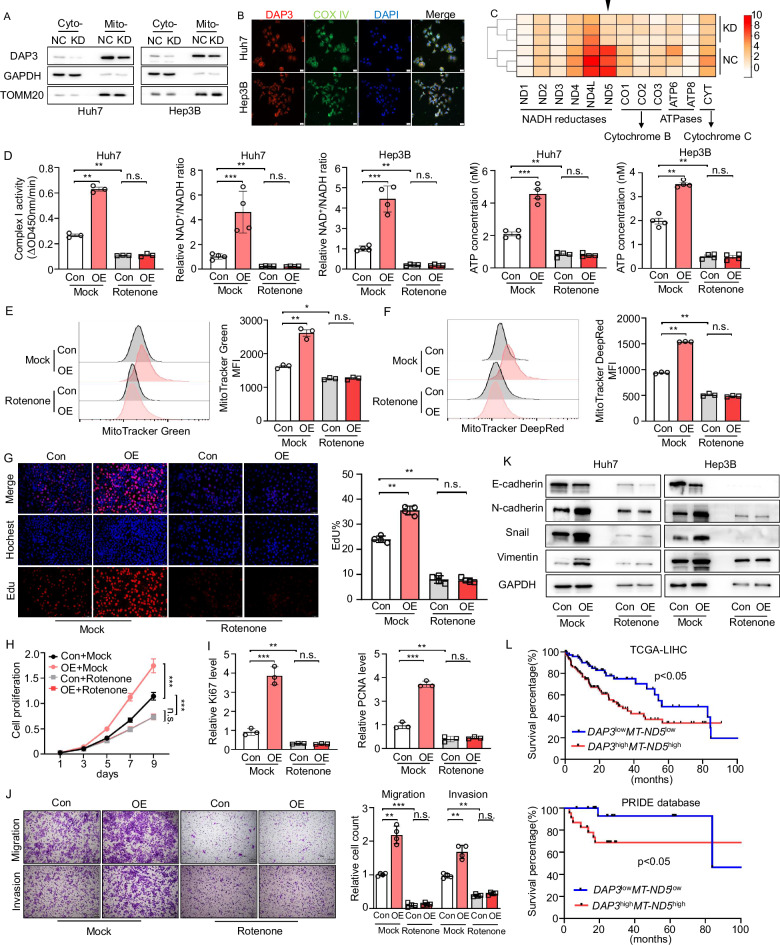
DAP3 modulates mitochondrial function and HCC progression by increasing mitochondrial complex I activity. **A** Western blot analysis of DAP3 expression in the cytoplasm and mitochondria. GAPDH was used as a loading control for cytoplasmic proteins, and TOMM20 was used as a loading control for mitochondrial proteins. **B** Immunofluorescence analysis of DAP3 colocalization with the mitochondrial marker protein COX IV in Huh7 and Hep3B cells (DAP3, red; COX IV, green; DAPI, blue). Scale bar, 20 μm. **C** Heatmap showing the analysis of the expression of 13 electron transfer chain proteins (encoded by the mitochondrial genome) in the DAP3-NC/KD Huh7 cells. **D**–**K** Huh7/Hep3B cells were transfected with the DAP3 overexpression vector or control vector for 48 hours and then treated with 100 nM rotenone for 24 h. **D** Analysis of ETC complex I enzyme activity (representative of three independent experiments). The NAD^+^/NADH ratio was determined by performing a colorimetric WST-8 assay (representative of three independent experiments), and ATP production was determined using an ATP Bioluminescent Assay Kit (representative of three independent experiments). **E**, **F** Flow cytometry analysis of the mitochondrial mass and mitochondrial membrane potential after labelling cells with MitoTracker Green (**E**) and MitoTracker DeepRed (**F**) (representative of three independent experiments). **G** EdU immunofluorescence staining (EdU, red; Hochest, blue) was performed to analyse the cell cycle after the indicated treatments and compare the percentage of cells in S phase. Scale bars, 50 μm. Hochest staining was used to detect total cells, and EdU staining indicated cells with active DNA replication. **H** CCK-8 assays were used to evaluate the proliferative capacity of Huh7 cells after the indicated treatments (representative of three independent experiments). **I** qPCR analysis of proliferation-related genes (Ki67 and PCNA) after the indicated treatments (representative of three independent experiments). **J** Transwell assays were used to measure the migration and invasion of Huh7 cells after the indicated treatments. Scale bars, 100 μm. Bar charts showing the relative counts (relative to the NC group) of cells that passed through the chamber membrane in each group (right panel) (representative of three independent experiments). **K** Western blot analysis of EMT-related proteins (E-cadherin, N-cadherin, Snail, and Vimentin) in Huh7 and Hep3B cells subjected to the indicated treatments (representative of three independent experiments). **L** OS analysis of HCC patients with different levels of DAP3 and MT-ND5 mRNA expression. The data were obtained from TCGA-LIHC database and the PRIDE database PXD006512. Statistical analysis was performed using two-way ANOVA (Fig. 4H), 2-tailed Student’s t test (Fig. 4D, E, F, G, I, and J), or the log-rank (Mantel‒Cox) test (Fig. 4L). The error bars indicate the means ± SDs. n.s., not significant; *p < 0.05; **p < 0.01; ***p < 0.001; and ****p < 0.0001.

We first confirmed the mitochondrial localization of DAP3 in HCC cells to investigate the mechanism by which DAP3 regulates mitochondrial function and HCC progression (Fig. 4A, B), and the results were consistent with the findings of previous studies [12]. A proteomic analysis was subsequently performed to examine the expression of 13 mtDNA-encoded proteins in the DAP3-NC/KD Huh7 cells. The analysis of the proteomic data revealed that MT-ND5, whose expression level is increased in HCC tissues and has a positive correlation with the DAP3 level, exhibited reduced expression in DAP3-KD Huh7 cells compared with control Huh7 cells [24] (Fig. 4C, Supplementary Fig. S4A, B).

We detected changes in MT-ND5 mRNA and protein expression in Huh7 and Hep3B cells with different DAP3 expression levels to clarify whether DAP3 regulates MT-ND5 expression at the transcriptional or translational level. We found that DAP3 could regulate the MT-ND5 protein level rather than the mRNA level in HCC cells (Supplementary Fig. S4C). More importantly, the introduction of DAP3 increased the MT-ND5 protein level only in control Huh7 cells and not in cells pretreated with CHX (Supplementary Fig. S4D). These results indicate that DAP3 participates in the translation of MT-ND5 in HCC cells.

As the largest component of the oxidative phosphorylation system, complex I is crucial for cellular respiration in many aerobic organisms [27–30]. MT-ND5 is one of the 14 evolutionarily conserved core subunits required for the catalytic function of complex I in the mitochondrial respiratory chain [31, 32]. We treated Huh7 cells with rotenone to determine whether DAP3 regulates mitochondrial activity and HCC progression through complex I. We found that the DAP3-induced increases in mitochondrial complex I activity, NAD^+^ generation, and ATP production could be inhibited by rotenone treatment (Fig. 4D). Consistently, the mitochondrial mass and mitochondrial membrane potential were significantly reduced after rotenone treatment (Fig. 4E, F). Similarly, the DAP3-induced increases in proliferation and Ki67 and PCNA expression were attenuated by rotenone treatment (Fig. 4G–I, Supplementary Fig. S4E). Moreover, rotenone-treated cells also exhibited reduced migration and invasion capacities (Fig. 4J). Further study revealed that rotenone treatment suppressed the DAP3-induced upregulation of N-cadherin, Vimentin, and Snail expression, as well as the decrease in E-cadherin expression (Fig. 4K). TCGA and PRIDE database showed that HCC patients with high DAP3 and MT-ND5 expression had a significantly worse prognosis than those with low DAP3 and MT-ND5 expression (Fig. 4L). These results revealed that DAP3 modulates mitochondrial function and HCC progression by regulating MT-ND5 translation and increasing mitochondrial complex I activity.

### DAP3^185S^ is a key site that modulates mitochondrial function and HCC progression

**Fig. 5:**
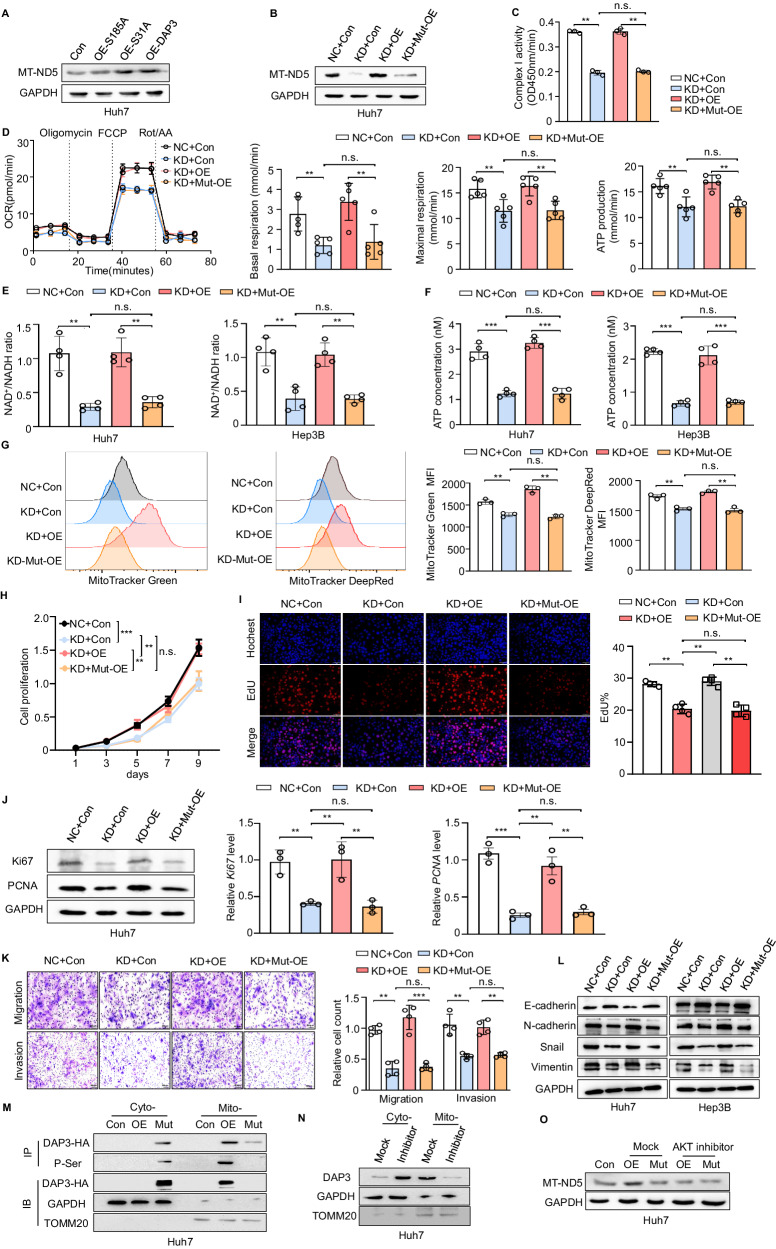
DAP3^185S^ is a key site that modulates mitochondrial function and HCC progression. **A** Huh7 cells were transfected with the DAP3 overexpression vector (OE-DAP3), DAP3^S185A^ overexpression vector (OE-S185A), DAP3^S31A^ overexpression vector (OE-S31A) or control vector (Con) for 48 hours. MT-ND5 protein expression was measured using western blotting. **B**–**L** Huh7/Hep3B cells with stable DAP3 knockdown (KD) and the corresponding negative control (NC) Huh7/Hep3B cells were transfected with the DAP3 overexpression vector (OE), DAP3^S185A^ overexpression vector (Mut-OE) or control vector (Con) for 48 h. **B** MT-ND5 protein expression was measured using western blotting. **C** Mitochondrial ETC complex I enzyme activity levels in the indicated groups (representative of three independent experiments). **D** Representative OCR measurements in the indicated groups upon the addition of oligomycin, the mitochondrial decoupler FCCP, and rotenone + antimycin. Basal and maximal respiration and ATP production were quantitatively calculated. **E** The NAD^+^/NADH ratio was determined by performing a colorimetric WST-8 assay (representative of three independent experiments). **F** ATP production was determined using an ATP Bioluminescent Assay Kit (representative of three independent experiments). **G** Flow cytometry analysis of the mitochondrial mass and mitochondrial membrane potential after labelling cells with MitoTracker Green and MitoTracker DeepRed (representative of three independent experiments). **H** CCK-8 assays were used to evaluate the proliferative capacity of Huh7 cells after the indicated treatments (representative of three independent experiments). **I** EdU incorporation assays (EdU, red; Hochest, blue) were performed to analyse the cell cycle after the indicated treatments and compare the percentage of S phase cells. Scale bars, 50 μm. Hoechst staining was used to indicate total cells, and EdU incorporation indicated cells with active DNA replication. **J** The relative expression of proliferation-related genes and proteins (Ki67 and PCNA) was verified using qPCR and western blotting (representative of three independent experiments). **K** Transwell assays were used to measure the migration and invasion of Huh7 cells after the indicated treatments. Scale bars, 200 μm. The bar charts show the relative counts (relative to the NC group) of cells that passed through the chamber membrane in each group (right panel) (representative of three independent experiments). **L** Western blot analysis of EMT-related proteins in Huh7 and Hep3B cells subjected to the indicated treatments (representative of three independent experiments). **M** The phosphorylation of wild-type or mutant DAP3 in the cytoplasm and mitochondria of Huh7 cells was detected using IP. GAPDH was used as a loading control for cytoplasmic proteins, and TOMM20 was used as a loading control for mitochondrial proteins. **N** Western blot analysis of DAP3 expression in the cytoplasm and mitochondria of cells treated with an AKT inhibitor (10 μmol/L for 24 h). **O** Western blot analysis of MT-ND5 expression in Huh7 cells transfected with OE-DAP3 or OE-S185A or treated with an AKT inhibitor (10 μmol/L for 24 hours). Statistical analysis was performed using two-way ANOVA (Fig. 5H) or 2-tailed Student’s t test (Fig. 5C, D, E, F, G, I, J, and K). The error bars indicate the means ± SDs. n.s., not significant; *p < 0.05; **p < 0.01; ***p < 0.001; and ****p < 0.0001.

DAP3 can be controlled by phosphorylation, serving as a molecular switch to impact protein synthesis and mitochondrial physiology [33]. The phosphorylation of DAP3 at Ser31 and Ser185 has been shown to decrease cell viability, and mutation of these phosphorylated residues may change DAP3 function. We transfected HCC cells with overexpression plasmids for the DAP3^S185A^ mutant (OE-S185A) and the DAP3^S31A^ mutant (OE-S31A) and then performed a western blot analysis of MT-ND5 expression to clarify the key sites of DAP3 involved in regulating MT-ND5 translation and expression. The MT-ND5 protein level was increased by the introduction of wild-type DAP3 or DAP3^S31A^ but not DAP3^S185A^ in HCC cells (Fig. 5A). Moreover, the DAP3-KD-induced decreases in MT-ND5 expression and mitochondrial complex I activity were reversed by DAP3 but not by DAP3^S185A^ overexpression (Fig. 5B, C). The Seahorse assay showed that DAP3^185S^ was the critical site that regulated mitochondrial basal and maximal respiration, ATP production, and NAD^+^ generation in HCC cells (Fig. 5D–F). In addition, the ability of DAP3 to regulate the mitochondrial mass and the mitochondrial membrane potential depended on its phosphorylation at Ser185 (Fig. 5G). These results revealed that DAP3^185S^ is a key site modulating MT-ND5 expression and mitochondrial function in HCC cells.

We next investigated the functional roles of DAP3^S185^ in HCC development. CCK8 and EdU incorporation assays showed that the DAP3-KD-induced suppression of cell proliferation was reversed by re-expressing DAP3 but not DAP3^S185A^ in Huh-7 cells (Fig. 5H, I). In addition, the DAP3-mediated regulation of Ki67 and PCNA expression was dependent on Ser185 (Fig. 5J). Moreover, DAP3^185S^ was found to be critical for regulating HCC cell invasion and migration, as well as the EMT process (Fig. 5K, L). Therefore, we concluded that DAP3 promoted HCC progression via its phosphorylation at Ser185.

As AKT-mediated phosphorylation of DAP3 at Ser/Thr residues can regulate the localization of DAP3 in mitochondria [17, 34], immunoprecipitation (IP) assays were further performed to examine the importance of phosphorylation at Ser185 for the mitochondrial localization of DAP3. Wild-type DAP3 was located mainly in mitochondria and exhibited serine phosphorylation, while the Ser185 mutant of DAP3 was retained in the cytosol and exhibited a significantly reduced level of serine phosphorylation (Fig. 5M). Treatment with the specific AKT inhibitor MK-2206 resulted in cytoplasmic retention of DAP3 and inhibited DAP3-induced MT-ND5 expression in Huh7 cells (Fig. 5N, O). These results indicate that the AKT-mediated phosphorylation of Ser185 is essential for the mitochondrial localization of DAP3 in HCC cells.

### DAP3^185S^ is a key site for the DAP3-mediated modulation of HCC cell senescence

**Fig. 6:**
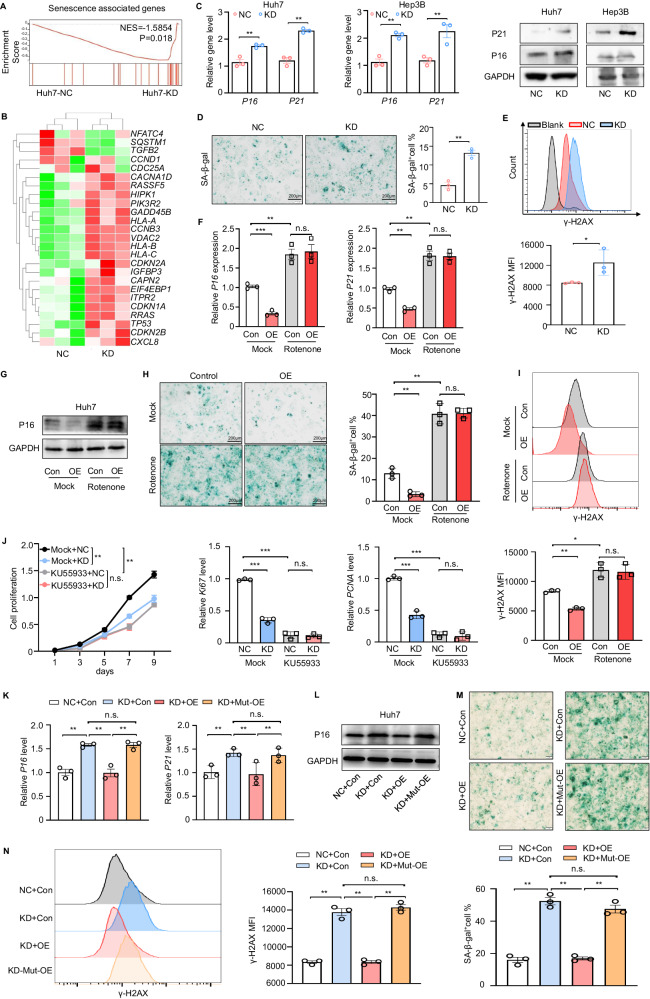
DAP3^185S^ is a key site for DAP3-mediated modulation of HCC cell senescence. **A** GSEA plots of the ranked list of differentially expressed genes in the KD and NC groups were generated using the senescence-associated gene set. **B** Heatmap indicating specific senescence-related gene expression patterns in the DAP3 NC/KD groups. **C**–**E** Huh7/Hep3B cells with stable DAP3 knockdown (KD) and the corresponding negative control (NC) cells were used to evaluate indicators of cellular senescence. **(C)** qPCR and western blot analyses of senescence-associated gene and protein expression in DAP3-NC/KD HCC cells (representative of three independent experiments). **D** Representative images of senescence-associated β-galactosidase (SA-β-gal) staining in NC and DAP3-KD Huh7 cells. Scale bars, 200 μm (representative of three independent experiments). Blue indicates positive staining. **E** Flow cytometry analysis of the MFI of the DNA damage marker γ-H2AX in NC or DAP3-KD Huh7 cells (representative of three independent experiments). **F**–**I** Huh7 cells were transfected with the DAP3 overexpression vector or control vector for 48 hours and were then treated with 100 nM rotenone for 24 hours. **F**, **G** qPCR (**F**) and western blot (**G**) analyses of senescence-associated gene and protein expression in the indicated groups (representative of three independent experiments). **H** Representative images of SA-β-gal staining (scale bars, 200 μm). Blue indicates positive staining (representative of three independent experiments). **I** Flow cytometry analysis of the γ-H2AX MFI (representative of three independent experiments). **J** Huh7/Hep3B cells with stable DAP3 knockdown (KD) and the corresponding negative control (NC) Huh7 cells were treated with 10 mg/mL KU55933 for 12 h. CCK-8 assays were used to evaluate the proliferative capacity of Huh7 cells after the indicated treatments (representative of three independent experiments), and qPCR analysis of proliferation-related genes (KI67 and PCNA) was performed after the indicated treatments (representative of three independent experiments). **K**–**N** Huh7/Hep3B cells with stable DAP3 knockdown (KD) and the corresponding negative control (NC) Huh7 cells were transfected with the DAP3 overexpression vector (OE), DAP3^S185A^ overexpression vector (Mut-OE) or control vector (Con) for 48 hours. **K**, **L** qPCR (**K**) and western blot (**L**) analyses of senescence-associated gene and protein (P16, P21) expression in the indicated groups (representative of three independent experiments). **M** Representative images of SA-β-gal staining. Scale bars, 200 μm. Blue indicates positive staining (representative of three independent experiments). **N** Flow cytometry analysis of the γ-H2AX MFI (representative of three independent experiments). Statistical analysis was performed using two-way ANOVA (Fig. 6J) or 2-tailed Student’s t test (Fig. 6C, D, E, F, H, I, J, K, M and N). The error bars indicate the means ± SDs. n.s., not significant; *p < 0.05; **p < 0.01; ***p < 0.001; and ****p < 0.0001.

As a potent tumour suppressor, senescence is thought to be both a cause and a consequence of mitochondrial dysfunction [35, 36]. Since DAP3 modulates mitochondrial function and metabolism, we further investigated whether DAP3 can control HCC cell senescence. According to the analysis of the TCGA database, the DNA repair, G2M checkpoint, and apoptosis scores were increased in the DAP3-high group (Supplementary Fig. S5A), and the expression of DAP3 in HCC samples was positively correlated with the expression of genetic maintenance- and cellular senescence-related genes [37] (Supplementary Fig. S5B, C). These results were consistent with our findings of the enrichment of senescence-related signatures in DAP3-KD Huh7 cells (Figs. 3A and 6A) and the upregulation of several senescence-related genes in the DAP3-KD group (Fig. 6B). DAP3-KD significantly increased the expression of senescence-related proteins such as P16 and P21 at both the transcriptional and translational levels (Fig. 6C). In addition, the percentage of SA-β-Gal (+) cells and the level of the DNA damage molecular marker γ-H2AX were significantly increased in DAP3-KD HCC cells (Fig. 6D, E).

We pretreated Huh7 cells with rotenone prior to DAP3 overexpression to identify the connection between DAP3-KD-induced mitochondrial dysfunction and HCC cell senescence. As shown in Fig. 6F, G, DAP3 overexpression inhibited P16 and P21 expression, while rotenone treatment not only increased P16 and P21 expression but also reversed the DAP3 OE-induced downregulation of P16 and P21 expression. In addition, SA-β-Gal staining and a flow cytometry analysis of γ-H2AX showed that rotenone reversed the DAP3-mediated antisenescence phenotype (Fig. 6H, I). The senescence-related ATM signalling pathway inhibitor KU55933 reversed the DAP3-KD-induced changes in cell proliferation and the expression of Ki67 and PCNA in HCC cells (Fig. 6J). These results indicate that DAP3 promotes the antisenescence phenotype of HCC cells by regulating mitochondrial function.

We next investigated whether Ser185 is a crucial site for regulating the antisenescence phenotype of HCC cells. We found that the DAP3 knockdown-induced upregulation of P16 and P21 was suppressed by the overexpression of wild-type DAP3 overexpression but not DAP3^S185A^ (Mut-OE) (Fig. 6K, L). SA-β-Gal staining and flow cytometry analyses of γ-H2AX further confirmed that wild-type DAP3 but not DAP3^S185A^ reversed the senescence phenotype of the DAP3-KD Huh7 cells (Fig. 6M, N). These results indicate that DAP3^185S^ is the key site for the regulation of the antisenescence phenotype of HCC cells.

## Discussion

Cellular metabolic reprogramming is a hallmark of cancer [38]. Mitochondria are hubs for metabolic reactions and drive metabolic reprogramming through multiple mechanisms. The functions of reprogrammed mitochondria support tumorigenesis through several pathways. For instance, excessive electron transport flux is found in cancer cells, which not only results in ATP production but also results in ROS formation [39]. These mitochondrial abnormalities are intimately related to dysregulated mitochondrial dynamics. A systematic functional evaluation of dysregulated mitochondrial proteins in cancer is still lacking. Here, by systematically analysing several databases of mitochondrial and total gene and protein expression in human HCC, we identified DAP3 as an important oncogene that regulates mitochondrial function and HCC progression. DAP3 expression in HCC was positively correlated with oxidative phosphorylation and the expression of mitochondrial function-related genes.

DAP3 is the only GTP-binding protein component of the small subunit of mammalian and yeast mitochondrial ribosomes [13, 40]. In yeast, DAP3 is required for mitochondrial DNA synthesis and respiration [41], indicating that DAP3 plays an important role in protein synthesis and is required for the maintenance of mitochondrial DNA. Our study also showed that DAP3 was localized to mitochondria and was overexpressed at the transcriptional and translational levels in HCC samples. DAP3-KD reduced mitochondrial respiration, cellular NAD^+^ and ATP levels, mitochondrial membrane potential, and cellular ROS levels. These reductions were accompanied by changes in mitochondrial size and shape, such as decreased mitochondrial length, mitochondrial fragmentation, and swollen cristae. DAP3 knockdown activated the UPRmt to combat mitochondrial dysfunction, thereby contributing to the recovery of the mitochondrial network.

**Fig. 7:**
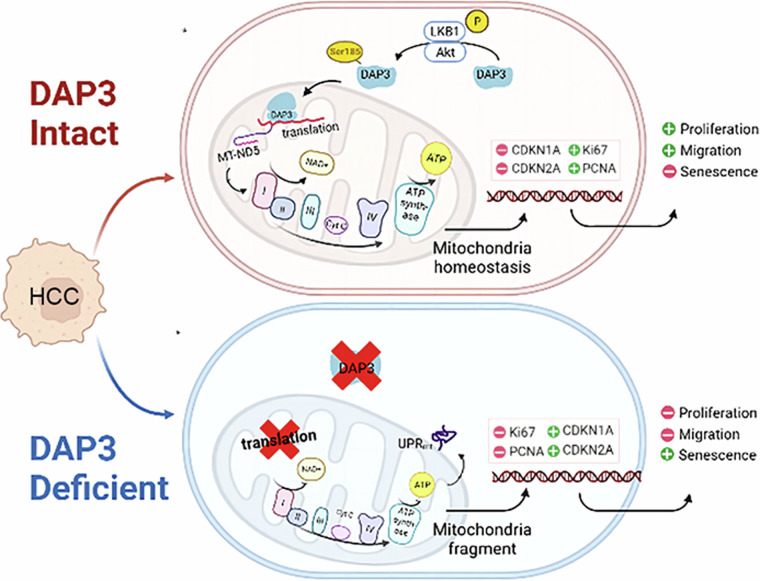
Schematic depicting the molecular mechanism by which DAP3 promotes mitochondrial function and HCC progression by regulating MT-ND5 expression.

Recently, the relationships between changes in mitochondrial function and the processes of cellular carcinogenesis and senescence have become increasingly clear. However, the roles of DAP3 in modulating mitochondrial function and HCC progression remain unclear. We found that the upregulation of DAP3 was associated with increased proliferation, migration, invasion, and metastasis of HCC cells, as well as with the EMT and increased levels of senescence-associated molecules. More importantly, the level and subcellular localization of DAP3 was associated with the antisenescence phenotype of HCC cells and could serve as important prognostic factors for HCC. Further study revealed that DAP3 promoted HCC progression and the cellular antisenescence phenotype by regulating the expression of MT-ND5, a component of mitochondrial complex I (Fig. 7). These results are consistent with the finding of Han et al. that DAP3 is a potent oncogene. However, Han et al. also showed that DAP3 functions by repressing RNA editing to negatively regulate the RNA editome by interacting with ADAR2, which is localized in the nucleus [15]. This result is different from our finding of the mitochondrial subcellular localization of DAP3 in HCC cells.

Mitochondria are highly dynamic, and the phosphorylation of Ser, Thr, or Tyr residues is possibly one of the most important regulatory mechanisms occurring in these organelles [15]. A previous study showed that DAP3 can be phosphorylated by AKT in osteosarcoma cells. The phosphorylation status of the mitochondrial ribosomal protein DAP3 is important for its translocation into mitochondria. We examined the subcellular localization of DAP3 in HCC cells and found that AKT-induced phosphorylation of DAP3 at Ser185 was critical for its mitochondrial localization. Blockade of AKT kinase activity or mutation of DAP3 at Ser185 caused the sequestration of DAP3 in the cytoplasm, resulting in its inability to perform translational functions, maintain mitochondrial function and metabolism, and maintain the invasive and antisenescence phenotype of HCC cells.

In conclusion, our study systematically investigated the dysregulated expression of mitochondrial genes and proteins in HCC and identified DAP3 as a potential target that regulates mitochondrial function and HCC progression. DAP3 could drive the malignant properties and antisenescence phenotype of HCC cells by regulating MT-ND5 expression. Our study contributes to the understanding of mitochondrial dynamics-induced HCC progression and provides strong evidence for a novel strategy for HCC treatment by targeting DAP3. However, the molecular mechanism by which DAP3 regulates MT-ND5 expression needs to be further explored.

### Supplementary information

Supplementary Materials

Original Data

## Data Availability

The data that support the findings of this study are available in the supplementary material of this article. The original western blot data has also been placed in the supplementary material.
