# Supplementary material for: DAP3 promotes mitochondrial activity and tumour progression in hepatocellular carcinoma by regulating MT-ND5 expression
Source: Cell Death Dis. 2024 Jul 29;15(7):540. doi: 10.1038/s41419-024-06912-2 (PMC11289107; doi:10.1038/s41419-024-06912-2)
Supplement: Supplementary file 1 — Supplementary Materials [file 41419_2024_6912_MOESM1_ESM.docx]

**Supplementary Materials**

**
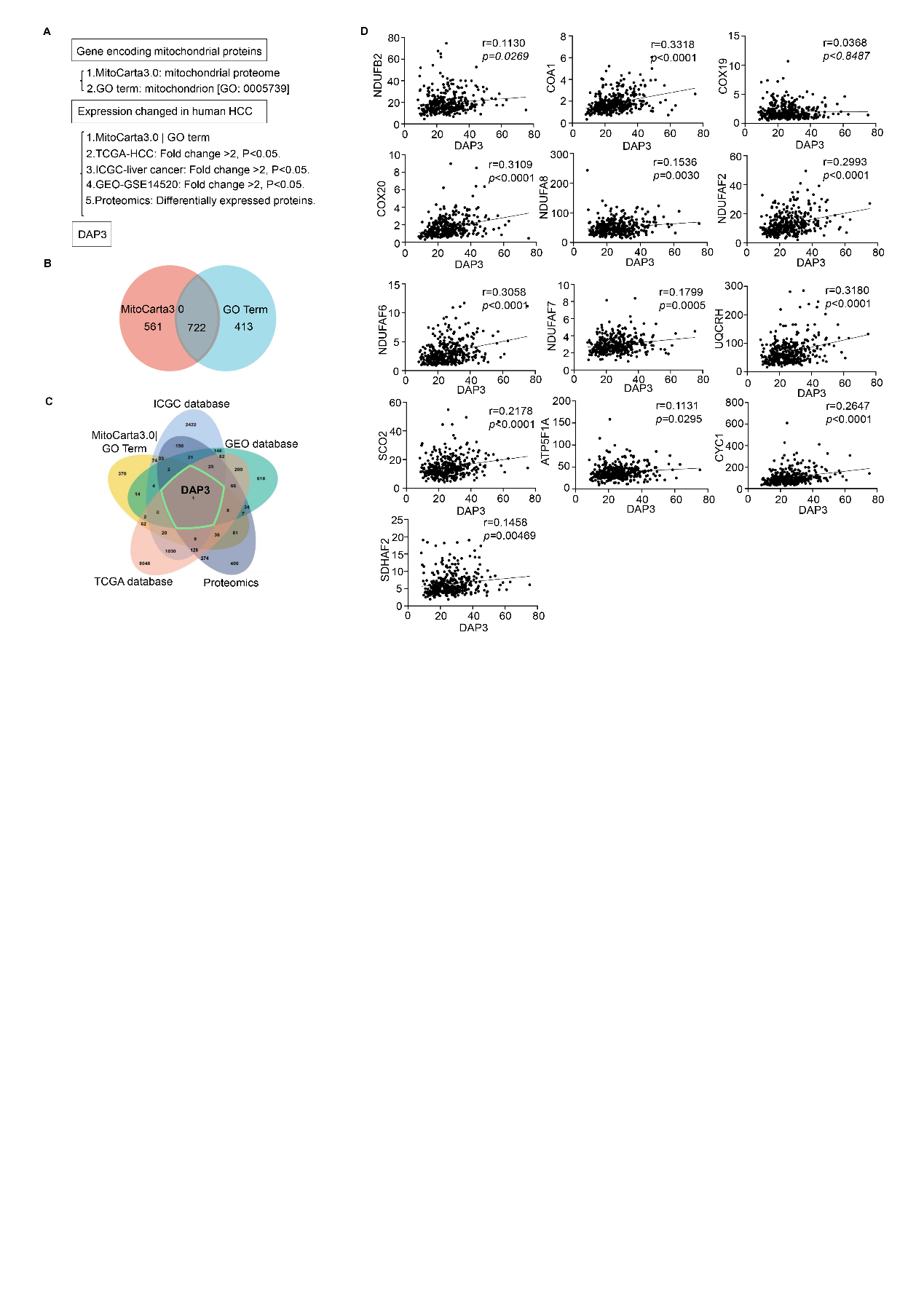
**

**Supplementary Fig. S1 Screening for dysregulated expression of mitochondrial regulators in HCC.**

**(A)** Flowchart of the screen for dysregulated mitochondrial proteins in HCC. **(B)** Venn diagram showing shared mitochondrial-regulated genes and proteins in the MitoCarta 3.0 and Gene Ontology (GO) term databases. **(C)** Venn diagram showing the expression of dysregulated mitochondrial regulators in HCC. **(D)** Relative gene expression ratio in the *DAP3*-Low and *DAP3*-High groups. (n = 186 in the *DAP3*-Low group, n = 187 in the *DAP3*-High group).

**
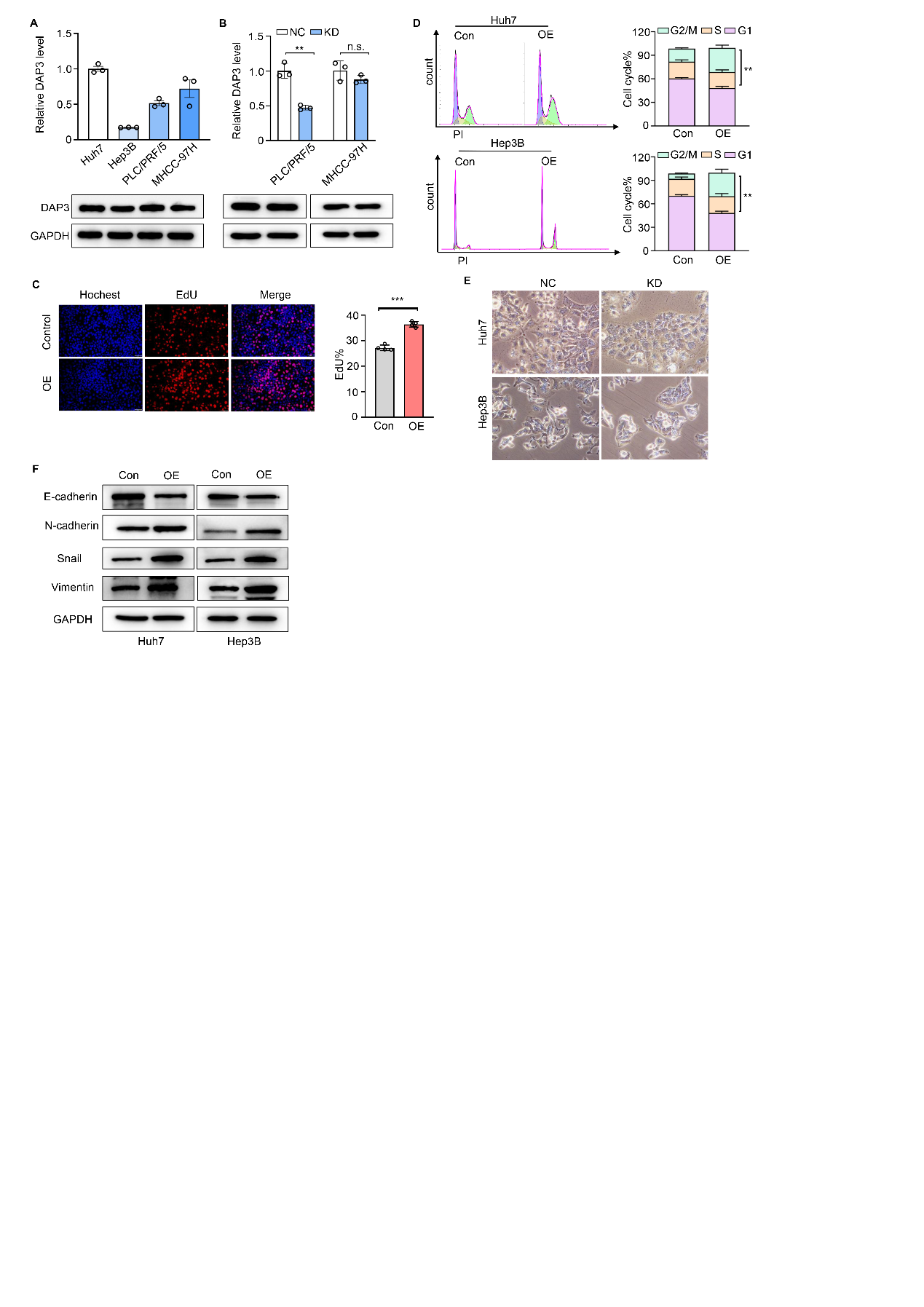
**

**Supplementary Fig. S2 DAP3 promotes HCC cell proliferation, migration, and invasion**.

**(A)** Relative mRNA and protein expression of DAP3 in HCC tumor cell lines (Huh7, Hep3B, PLC/PRF/5 and MHCC-97H) obtained by quantitative real‐time qPCR and western blotting assays (representative of three independent experiments). **(B)** The knockdown efficiency of *DAP3* in MHCC-97H and PLC/PRF/5 cells was verified by qRT‐PCR and western blotting assays. **(C)** EdU immunofluorescence staining assays (EdU, red; DAPI, blue) were conducted in HCC cells after overexpression of DAP3 (Scale bar: 200 μm). **(D)** The cell cycle in Huh7 and Hep3B cells after overexpression of DAP3 was detected by flow cytometry. **(E)** The cellular morphology of Huh7/Hep3B cells with low DAP3 expression was observed by microscopy. **(F)** Expression of EMT-related proteins in Huh7 and Hep3B cell lines with DAP3 overexpression. n.s., *no significance*; *, *p* < 0.05; **, *p* < 0.01; ***, *p* < 0.001; ****, *p* < 0.0001.

**
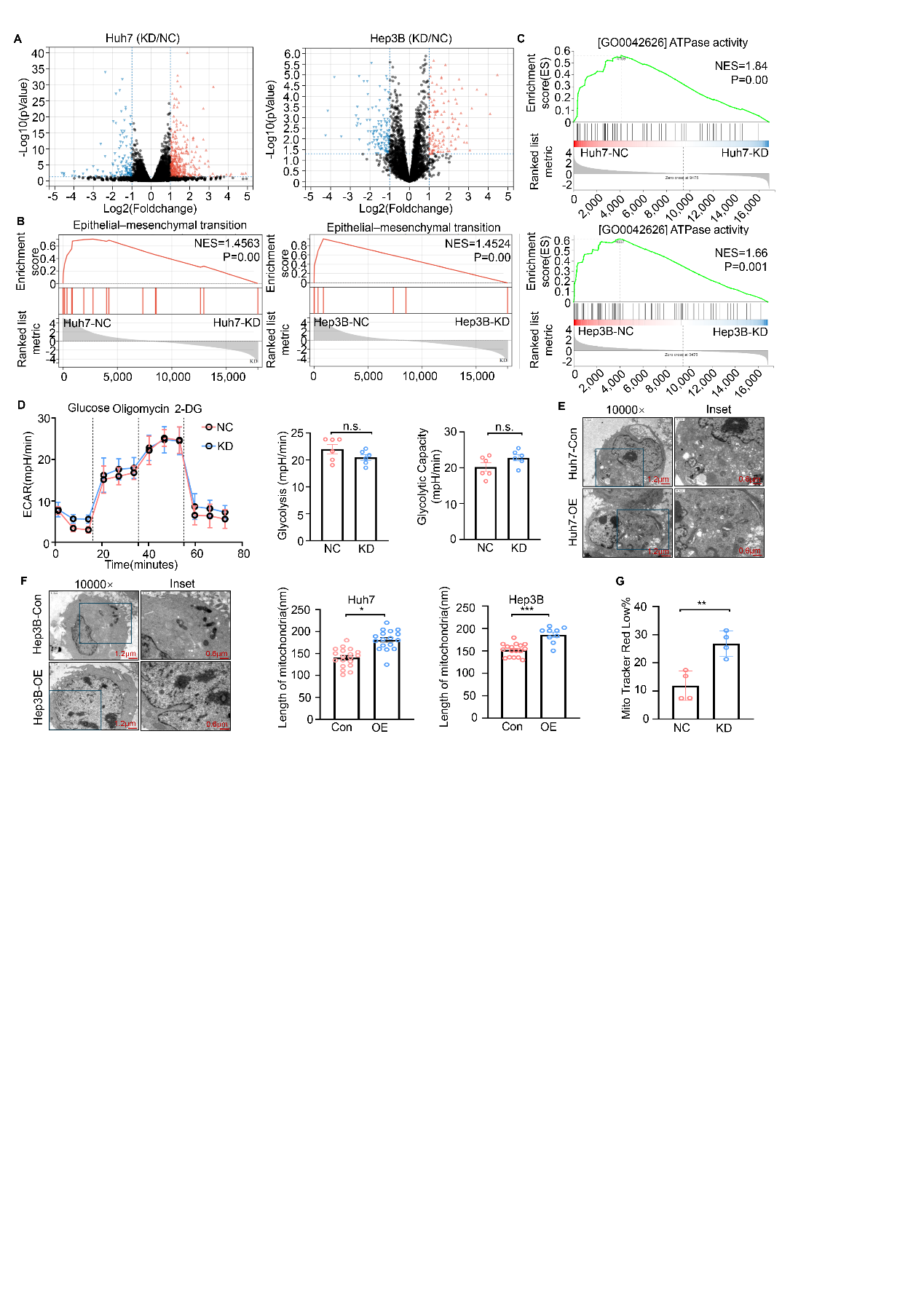
**

**Supplementary Fig. S3.** **DAP3 improves HCC mitochondrial homeostasis and reprograms metabolism.**

**(A)** Volcano plot (log2 [fold change] versus −log10 [P value]) showing differentially expressed genes in the *DAP3*-KD and DAP3-NC groups in Huh7 or Hep3B cells. **(B)** GSEA of Epithelial-mesenchymal transition in DAP3-NC Huh7 cells (left) or Hep3B cells (right) compared with KD cells. **(C)** GSEA plots of the ranked list of differentially expressed genes in KD and NC Huh7/Hep3B cells generated using ATPase activity. **(D)** Representative ECAR analysis of *DAP3* NC/KD-Huh7 cells upon the addition of glucose, oligomycin, and 2-DG. Quantified glycolysis and glycolytic capacity were calculated. **(E-F)** TEM images and statistical graphs showing the mitochondrial morphology in *DAP3* Con/OE-Huh7 or Hep3B cells. Scale bars, 1.2 μm (10000×), 0.6 μm (inset). **(G)** FACS analysis of the mitochondrial membrane potential of DAP3 NC/KD Huh7 cells. Bar charts show the relative MitoTracker Red-Low cell counts. n.s., *no significance*; *, *p* < 0.05; **, *p* < 0.01; ***, *p* < 0.001; ****, *p* < 0.0001.

**
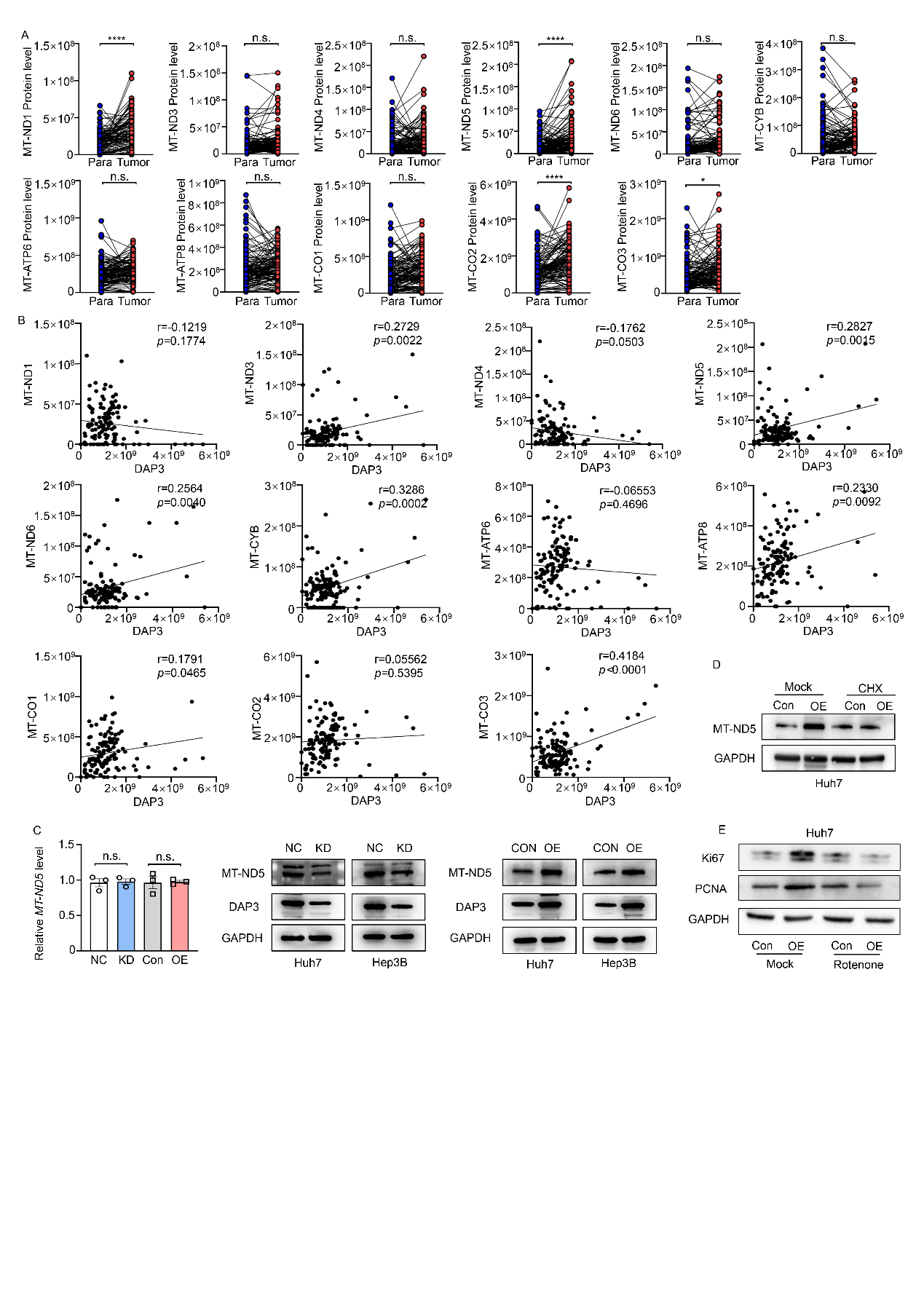
**

**Supplementary Fig. S4. MT-ND5 expression is upregulated in HCC cells and positively correlates with DAP3 expression.**

**(A)** Analysis of OXPHOS complex protein levels in HCC tumor tissues compared to para-tumor tissues. Data were obtained from the PRIDE database ([www.ebi.ac.uk/pride/archive](http://www.ebi.ac.uk/pride/archive), accession numbers PXD006512 and PXD008373). Quantification for the detection of endogenous MT-ND2 and MT-ND4L could not be validated in the proteomics data. (B) Analysis of the relationship between OXPHOS complex protein and DAP3 expression. Data were obtained from the PRIDE database ([www.ebi.ac.uk/pride/archive](http://www.ebi.ac.uk/pride/archive), accession numbers PXD006512 and PXD008373). **(C)** The relative mRNA and protein expression levels of MT-ND5 were measured by qPCR and western blot analysis in DAP3-OE or DAP3-KD Huh7 cells (representative of three independent experiments). **(D)** Expression of MT-ND5 in Huh7 cells which were transfected with DAP3 overexpression (OE) or control vector (Con) for 48 hours and then treated with 10 μg/mL CHX for 8 hours. **(E)** Western blotting assays of proliferation-related genes (Ki67, PCNA) with the indicated treatment. n.s., *no significance*; *, *p* < 0.05; **, *p* < 0.01; ***, *p* < 0.001; ****, *p* < 0.0001.

**
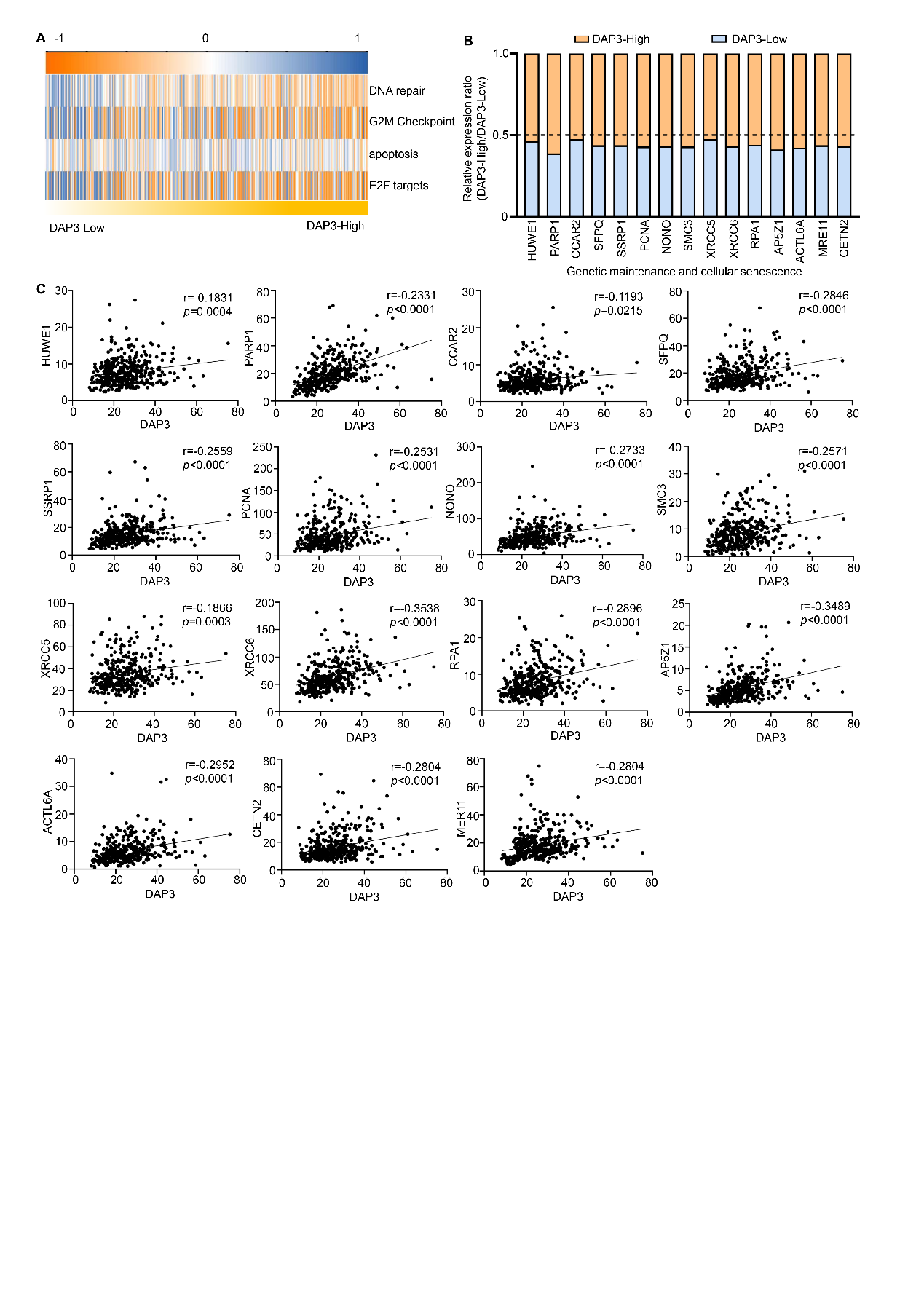
**

**Supplementary Fig. S5. DAP3 expression is associated with genetic maintenance genes.**

1. GSVA analysis of DAP3 expression with cell proliferation, DNA repair, and recombination. Data were obtained from the TCGA database. **(B)** Relative gene expression ratio in the *DAP3*-Low and *DAP3*-High groups. **(C)** Analysis of the relationship between *DAP3* and senescence-related genes (*HUWE1, PARP1, CCAR2, SFPQ, SSRP1, PCNA, NONO, SMC3, XRCC5, XRCC6, RP1A, AP5Z1, ACTL6A, MRE11 and CETN2*) expression in HCC.

**Table. S1 Demographic and clinical characteristics of patients.**

| **Liver Cancer patients (n=30)** | |
| --- | --- |
| Pathological diagnosis | HCC (n=30) |
| Age (years) | 56.8(11.8) |
| Gender (M/F) | (28/2) |
| Personal history of HBV | 60% (18/30) |
| AFP (ng/mL) | 36.67% (11/30) |
| Cirrhosis | 56.67% (17/30) |
| Tumor size (cm, ≤5/＞5) | (10/20) |
| Tumor differentiation (I+II/III+IV) | (5/7) |

**Table. S2 Reagent and Resource.**

| Reagent or Resource | Source | Identifier |
| --- | --- | --- |
| Antibodies |  |  |
| Anti-GAPDH antibody | HUABIO | Cat#ET1601-4 |
| Anti-GAPDH antibody | Proteintech | Cat#60004-1-lg |
| Anti-β-actin antibody | Proteintech | Cat#66009-1-Ig |
| Anti-DAP3-antibody | Thermo Fisher Scientific | Cat#MA5-26568 |
| Anti-DAP3 antibody | Abcam | Cat#ab227257 |
| Anti-TOMM20 antibody | Abcam | Cat#ab186735 |
| Anti-MT-ND5 antibody | Abcam | Cat#ab230509 |
| Anti-Anti-Phospho - (Ser/Thr) Phe antibody | Abcam | Cat#ab17464 |
| Anti-E-Cadherin antibody | Cell Signaling Technology | Cat#14472 |
| Anti-E-Cadherin antibody | Cell Signaling Technology | Cat#13116 |
| Anti-Vimentin antibody | Cell Signaling Technology | Cat#5741 |
| Anti-Snail antibody | Cell Signaling Technology | Cat#3879 |
| Anti-Ki67 antibody | Abcam | Cat#ab16667 |
| Anti-PCNA antibody | Abcam | Cat#ab92552 |
| Anti-P16 antibody | Abclonal | Cat#A11058 |
| Anti-P21 antibody | Abclonal | Cat#A1483 |
| Anti-COXIV antibody | Proteintech | Cat#66110-1-lg |
| Anti-HSPE1 antibody | Proteintech | Cat#16512-1-AP |
| Anti-CLPP antibody | Proteintech | Cat#15698-1-AP |
| Anti-DDIT3 antibody | Proteintech | Cat#66741-1-lg |
| Anti-ATF4 antibody | Proteintech | Cat#10835-1-AP |
| Anti-HSPD1 antibody | Proteintech | Cat#15282-1-AP |
| Anti-γ-H2AX antibody | Novus | Cat# NB100-384 |
| Chemicals |  |  |
| Diethylmitrosamine (DEN) | Sigma-Aldrich | Cat#N0756 |
| D-Luciferin potassium salt | Beyotime Biotechnology | Cat#ST196 |
| MitoTracker Green | Thermo Fisher Scientific | Cat#M7514 |
| MitoTracker DeepRed | Thermo Fisher Scientific | Cat#M22426 |
| MitoSOX | Thermo Fisher Scientific | Cat#36005 |
| Pim1/AKK1-IN-1 | MedChemExpress | Cat#HY-10371 |
| Cycloheximide (CHX) | MedChemExpress | Cat#HY-12320 |
| KU-55933 | MedChemExpress | Cat#HY-12016 |
| Actinomycin D (ActD) | MedChemExpress | Cat#HY-17559 |
| Critical commercial assays | | |
| BD Pharmingen™ Cell Cycle Kit | BD Bioscience | Cat#558662 |
| BeyoClick™ EdU Cell Proliferation Kit with Alexa Fluor 594 | Beyotime Biotechnology | Cat#C0078L |
| Seahorse XF Mitochondrial Stress Assay Kit | Agilent Technologies | Cat# 103015-100 |
| Seahorse XF Glycolysis Stress Test Profile | Agilent Technologies | Cat#103344-100 |
| NAD+/NADH Assay Kit | Abcam | Cat#ab65348 |
| ATP Colorimetric/Fluorometric Assay Kit | Sigma-Aldrich | Cat#MAK190 |
| Complex I Enzyme Activity Microplate Assay Kit | Abcam | Cat#ab109721 |
| Cell Mitochondria Isolation Kit | Beyotime Biotechnology | Cat#C3601 |
| In Situ β-galactosidase Staining Kit | Beyotime Biotechnology | Cat#RG0039 |
| DNA Damage Assay Kit by γ-H2AX Immunofluorescence | Beyotime Biotechnology | Cat#C2035S |
| Experimental models |  |  |
| Human Hepatocellular Carcinoma cell Huh7 | Li. lab | N/A |
| Human Hepatocellular carcinoma cell Hep3B | Li. lab | N/A |
| Human Hepatocellular carcinoma cell PLC/PRF/5 | Li. lab | N/A |
| Human Hepatocellular carcinoma cell MHCC-97H | Li. lab | N/A |
| BALB/c-Nude mice | Gempharmatech | N/A |
| C57BL/6 mice | Gempharmatech | N/A |
| Oligonucleotides (5’ to 3’) |  |  |
| Human-*ACTIN*-F | CAACGAATTTGGCTACAGCA | BioSune |
| Human-*ACTIN*-R | AGGGGTCTACATGGCAACTG | BioSune |
| Human-*DAP3*-F | CTTGCCTGATGGTAAGGAAACC | BioSune |
| Human-*DAP3*-R | TGCAGAAGATCCCGACAATTTTT | BioSune |
| Human-*HSPD1*-F | GTGTAGACCTTTTAGCCGATGC | BioSune |
| Human-*HSPD1*-R | GTGCCAGTACAGTAGCAGTGG | BioSune |
| Human-*CDKN1A*-F | CGATGGAACTTCGACTTTGTCA | BioSune |
| Human-*CDKN1A*-R | GCACAAGGGTACAAGACAGTG | BioSune |
| Human-*CDKN2A*-F | ATGGAGCCTTCGGCTGACT | BioSune |
| Human-*CDKN2A*-R | GTAACTATTCGGTGCGTTGGG | BioSune |
| Human-*FOXO4*-F | CCGGCAAAAGCTCTTGGTG | BioSune |
| Human-*FOXO4*-R | GGTCCACATATCGGCTTCTTCA | BioSune |
| Human-*HSPE1*-F | ATGGCAGGACAAGCGTTTAGA | BioSune |
| Human-*HSPE1*-R | CCGATCCAACAGCGACTACT | BioSune |
| Human*-CLPP*-F | TTGCCAGCCTTGTTATCGCA | BioSune |
| Human-*CLPP*-R | GGTTGAGGATGTACTGCATCG | BioSune |
| Human-*UBL5*-F | GGGAAGAAGGTCCGCGTTAAA | BioSune |
| Human-*UBL5*-R | ACGTGGTCCTTAAAAATCGTGT | BioSune |
| Human-*DDIT3*-F | GGAAACAGAGTGGTCATTCCC | BioSune |
| Human-*DDIT3*-R | CTGCTTGAGCCGTTCATTCTC | BioSune |
| Human-*ATF4*-F | ATGACCGAAATGAGCTTCCTG | BioSune |
| Human-*ATF4*-F | GCTGGAGAACCCATGAGGT | BioSune |
| Software and algorithms |  |  |
| GraphPad Prism Software | GraphPad Prism | Version 8.3.0 |
| Flowjo | BD Biosciences | Version 10.7.2 |
| Image J Software | Softonic | N/A |
| DNAMAN | LynnonBiosoft | Version 6.0 |
| StrataQuest | TissueGnostics | Version 6.0 |
| R | The free software environment for statistical computing and graphics | N/A |
