# Supplementary material for: DAP3 promotes mitochondrial activity and tumour progression in hepatocellular carcinoma by regulating MT-ND5 expression
Source: Cell Death Dis. 2024 Jul 29;15(7):540. doi: 10.1038/s41419-024-06912-2 (PMC11289107; doi:10.1038/s41419-024-06912-2)

Fig. 1G

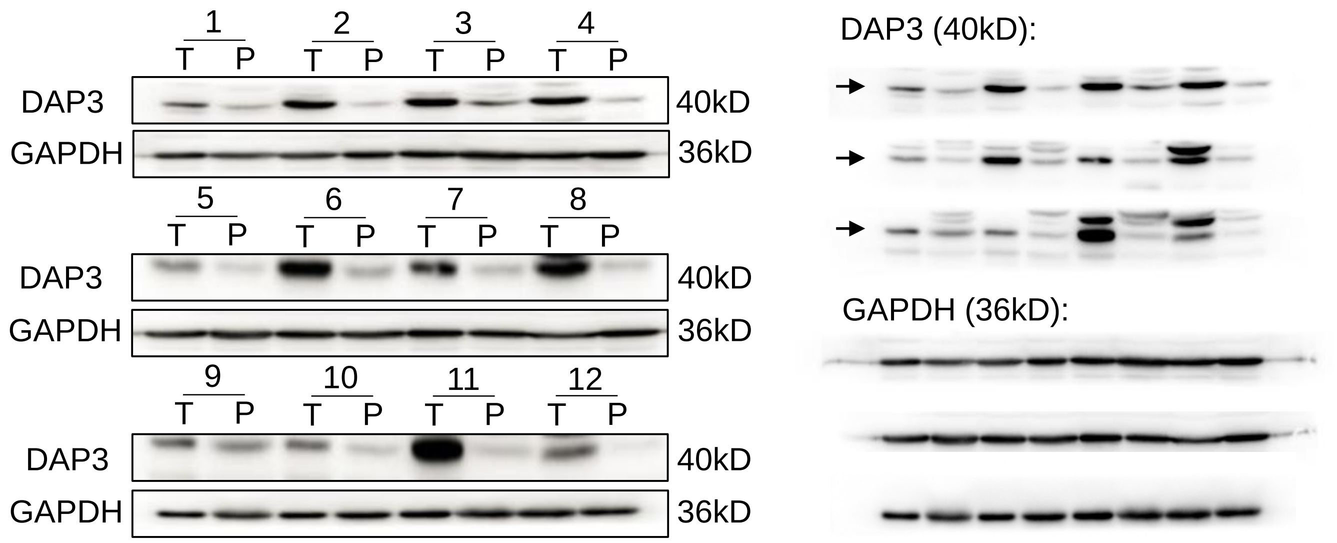

Fig. 1I

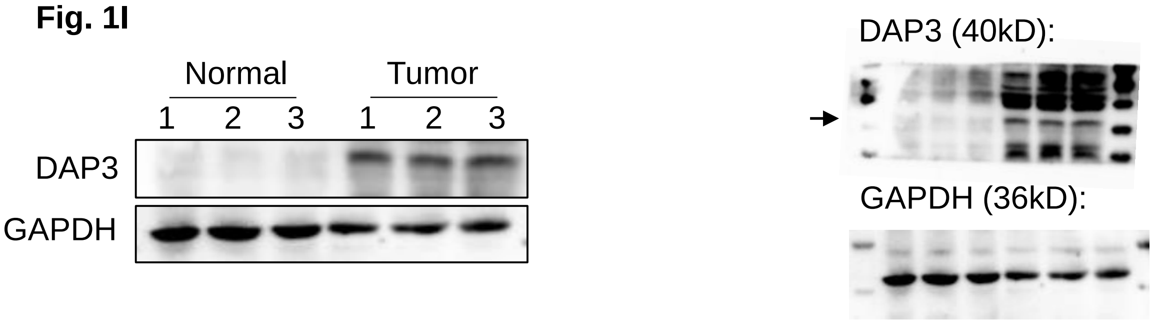

Fig. 1J

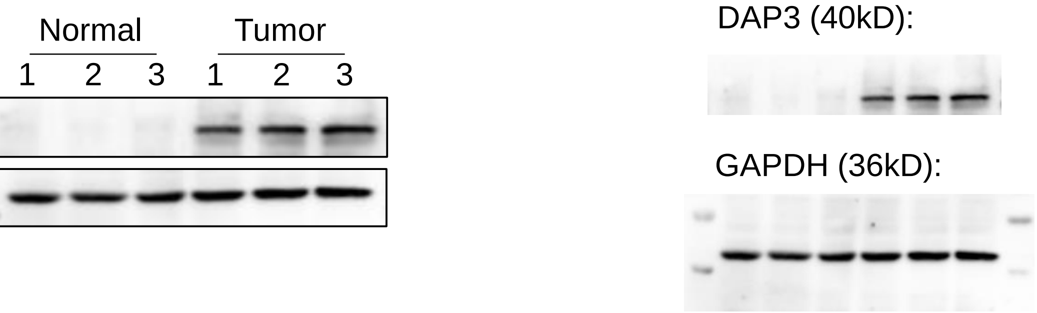

Fig. 2B

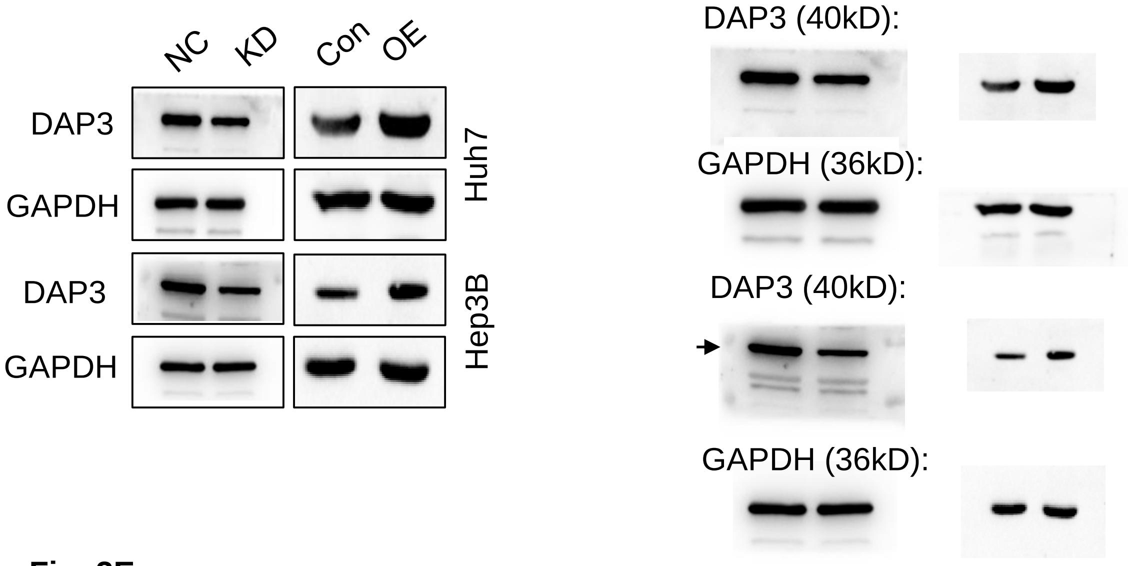

Fig. 2E

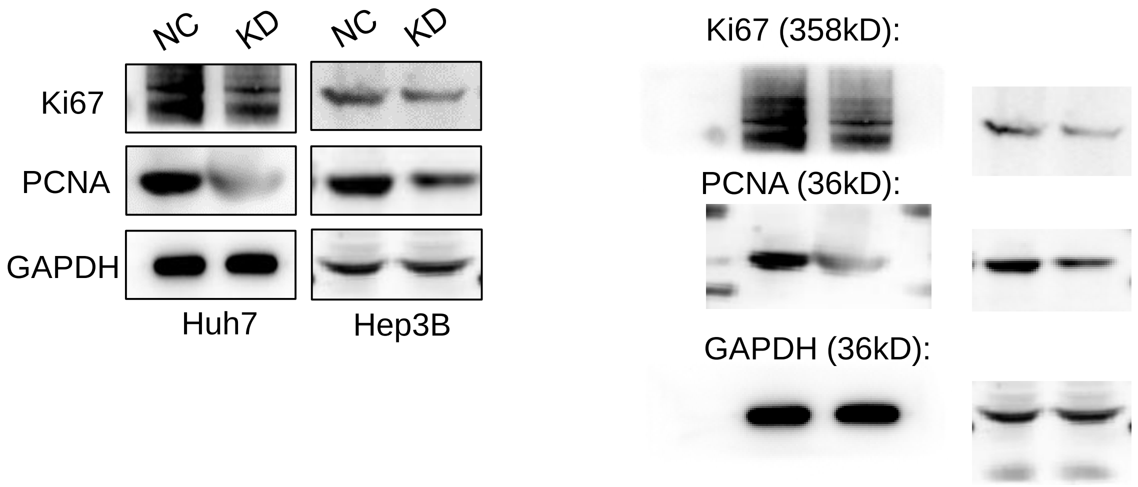

Fig. 2J

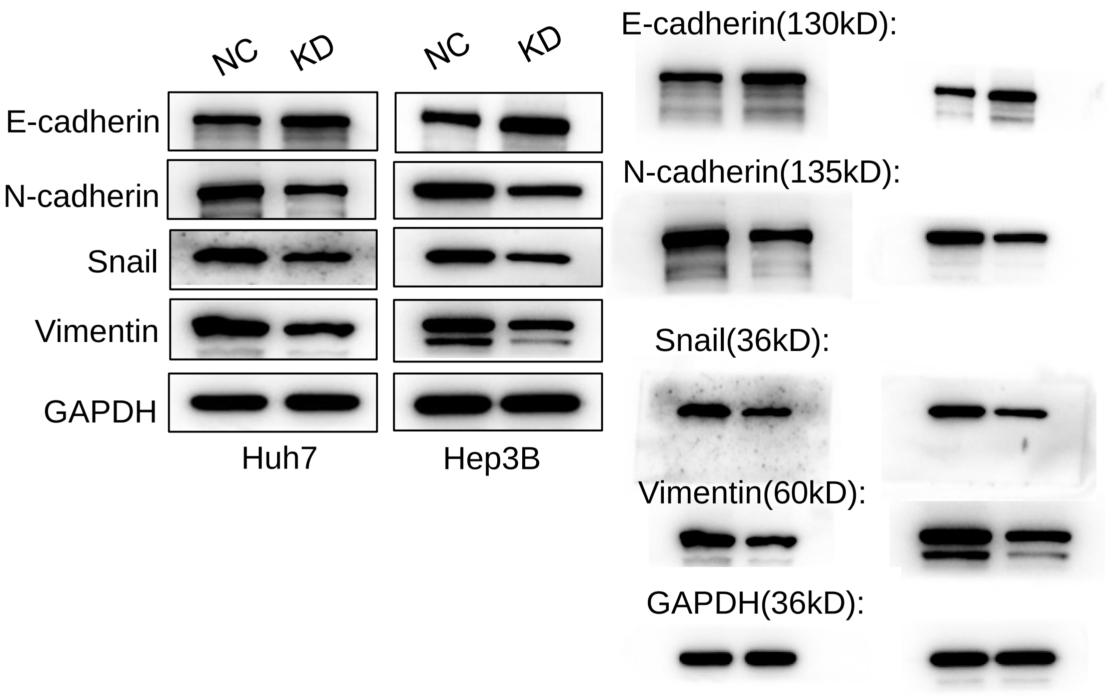

Fig. 3M

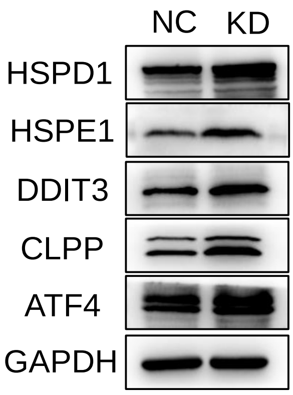
NC
KD
HSPD1
HSPE1
DDIT3
CLPP
ATF4
GAPDH

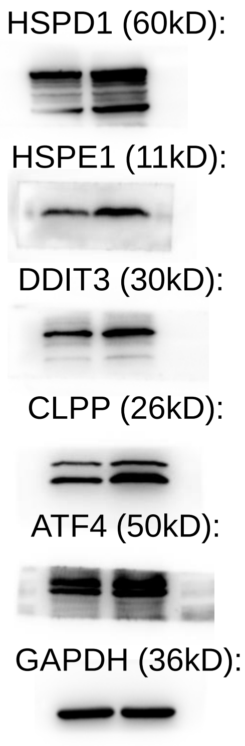
HSPD1 (60kD):
HSPE1 (11kD):
DDIT3 (30kD):
CLPP (26kD):
ATF4 (50kD):
GAPDH (36kD):

Fig. 4A

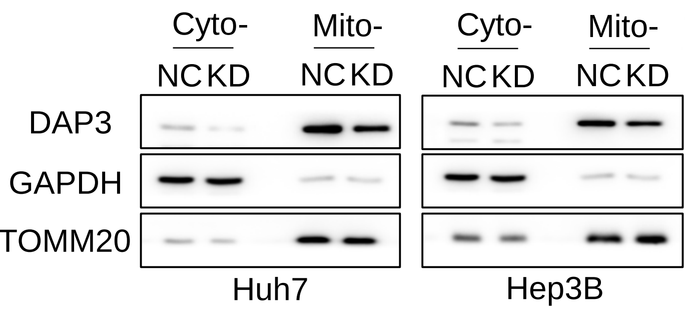
Cyto-
Mito-
Cyto-
Mito-
NC KD NC KD
NC KD NC KD
DAP3
GAPDH
TOMM20
Huh7
Hep3B

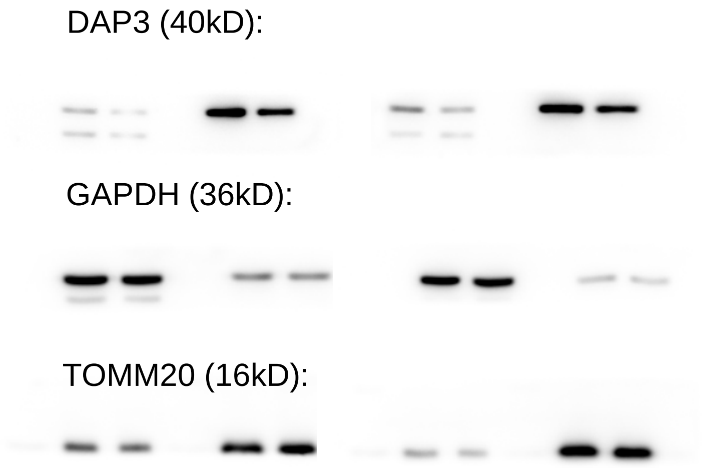
DAP3 (40kD):
GAPDH (36kD):
TOMM20 (16kD):

Fig. 4K

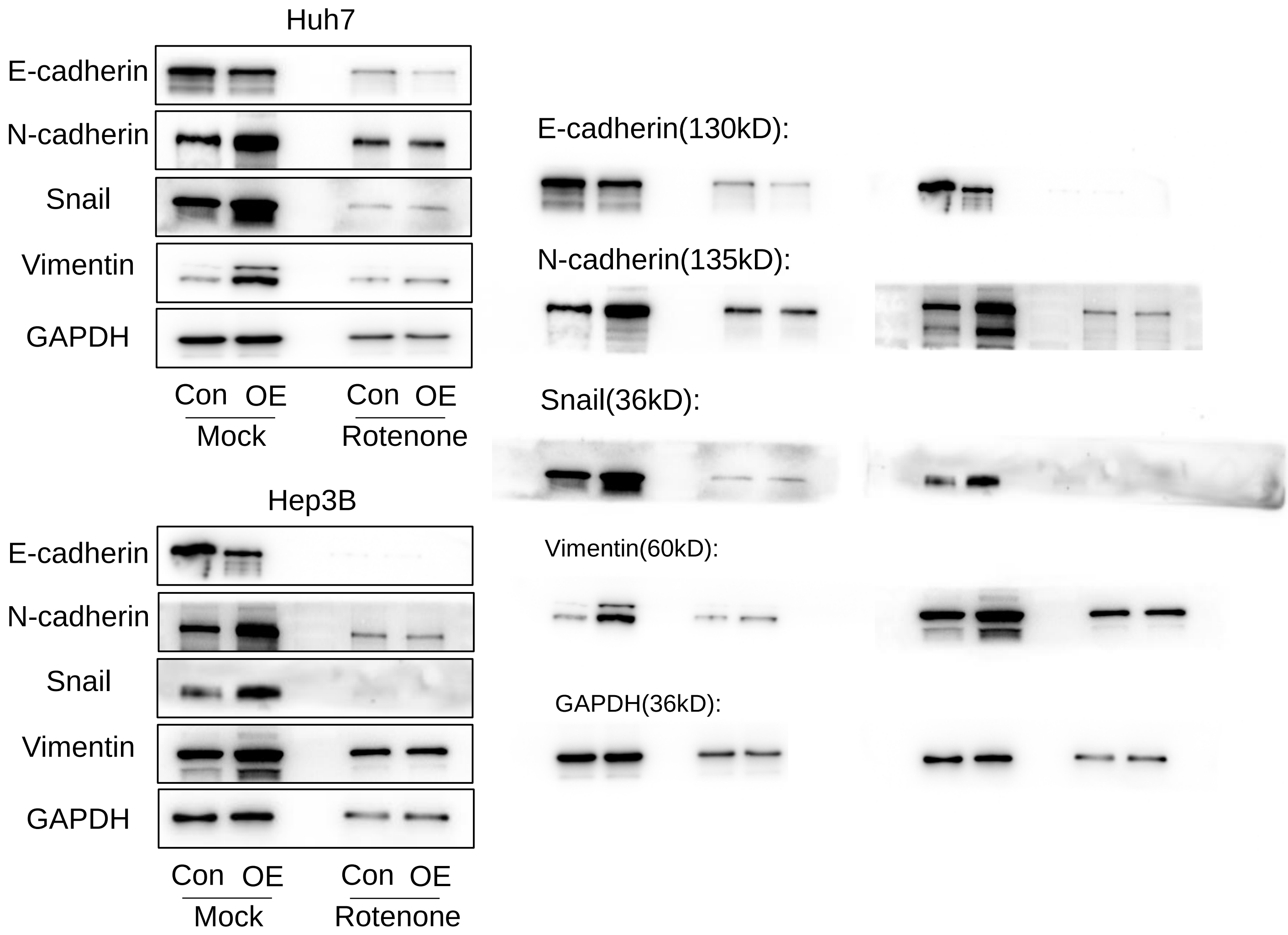
Huh7
E-cadherin
N-cadherin
Snail
Vimentin
GAPDH
Con OE
Mock
Con OE
Rotenone
Hep3B
E-cadherin
N-cadherin
Snail
Vimentin
GAPDH
Con OE
Mock
Con OE
Rotenone
E-cadherin(130kD):
N-cadherin(135kD):
Snail(36kD):
Vimentin(60kD):
GAPDH(36kD):

Fig. 5A

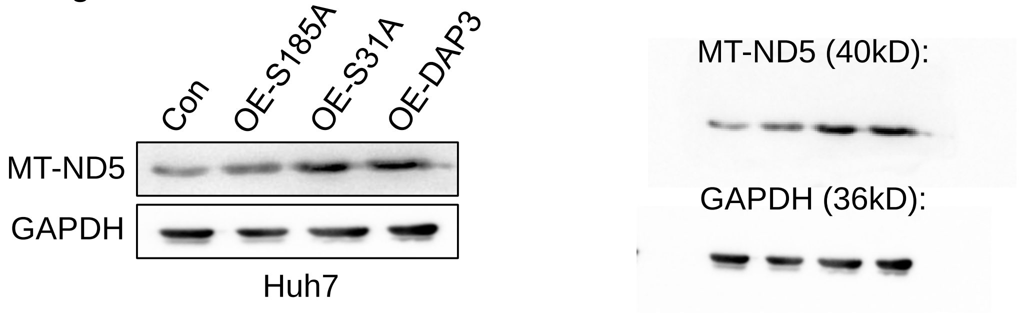

Fig. 5B

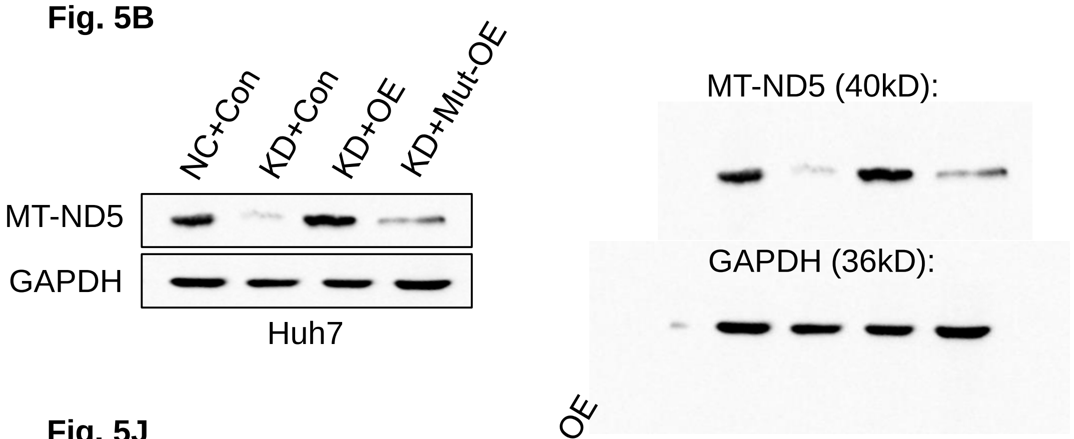

Fig. 5J

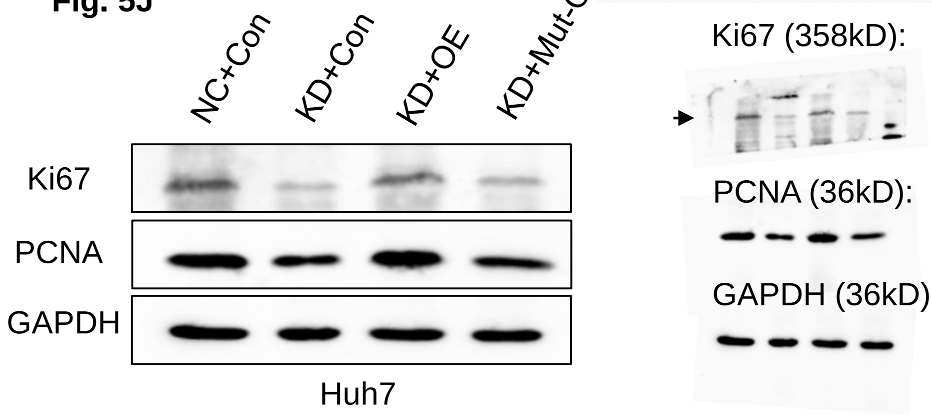

Fig. 5L

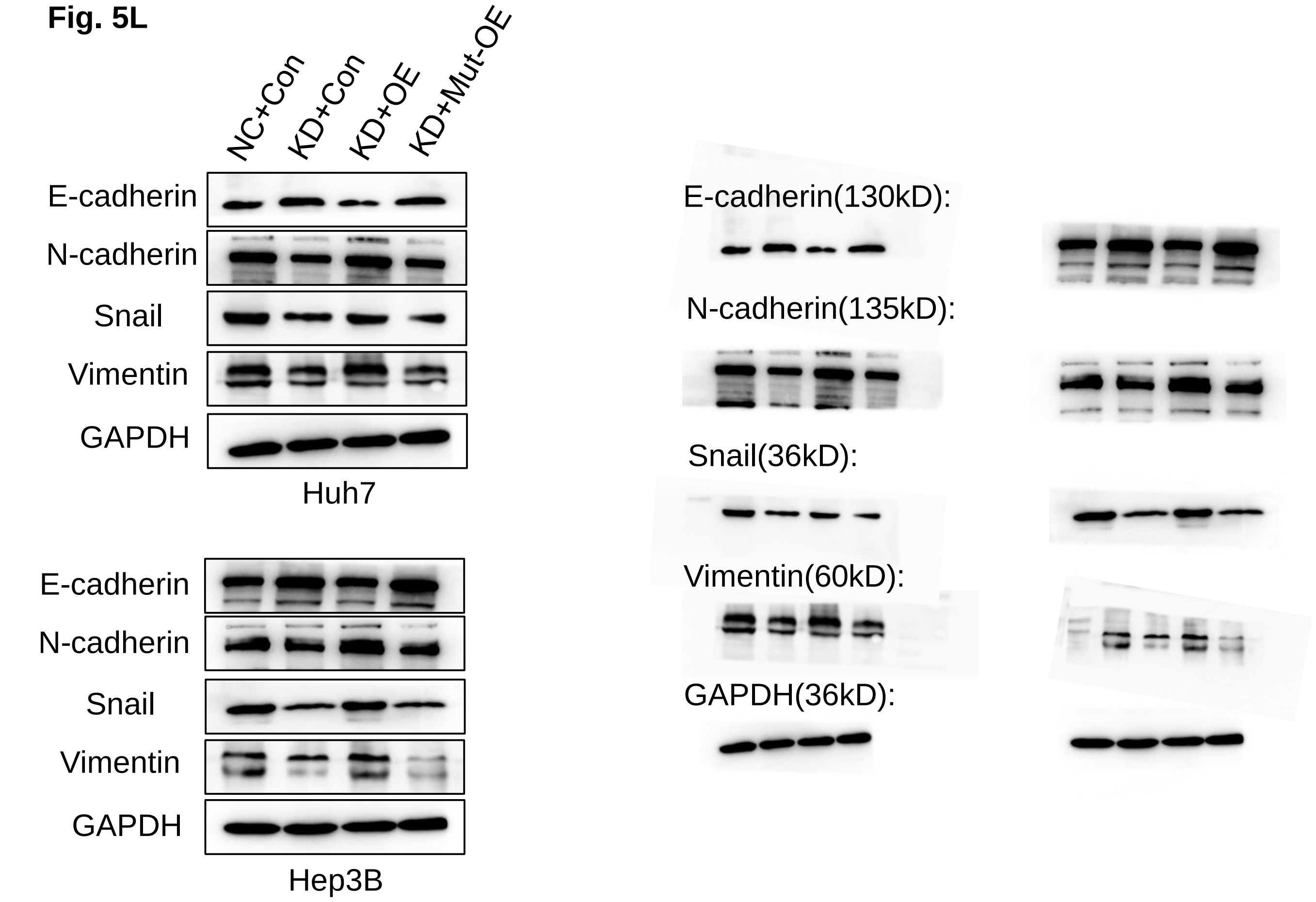

Fig. 5M

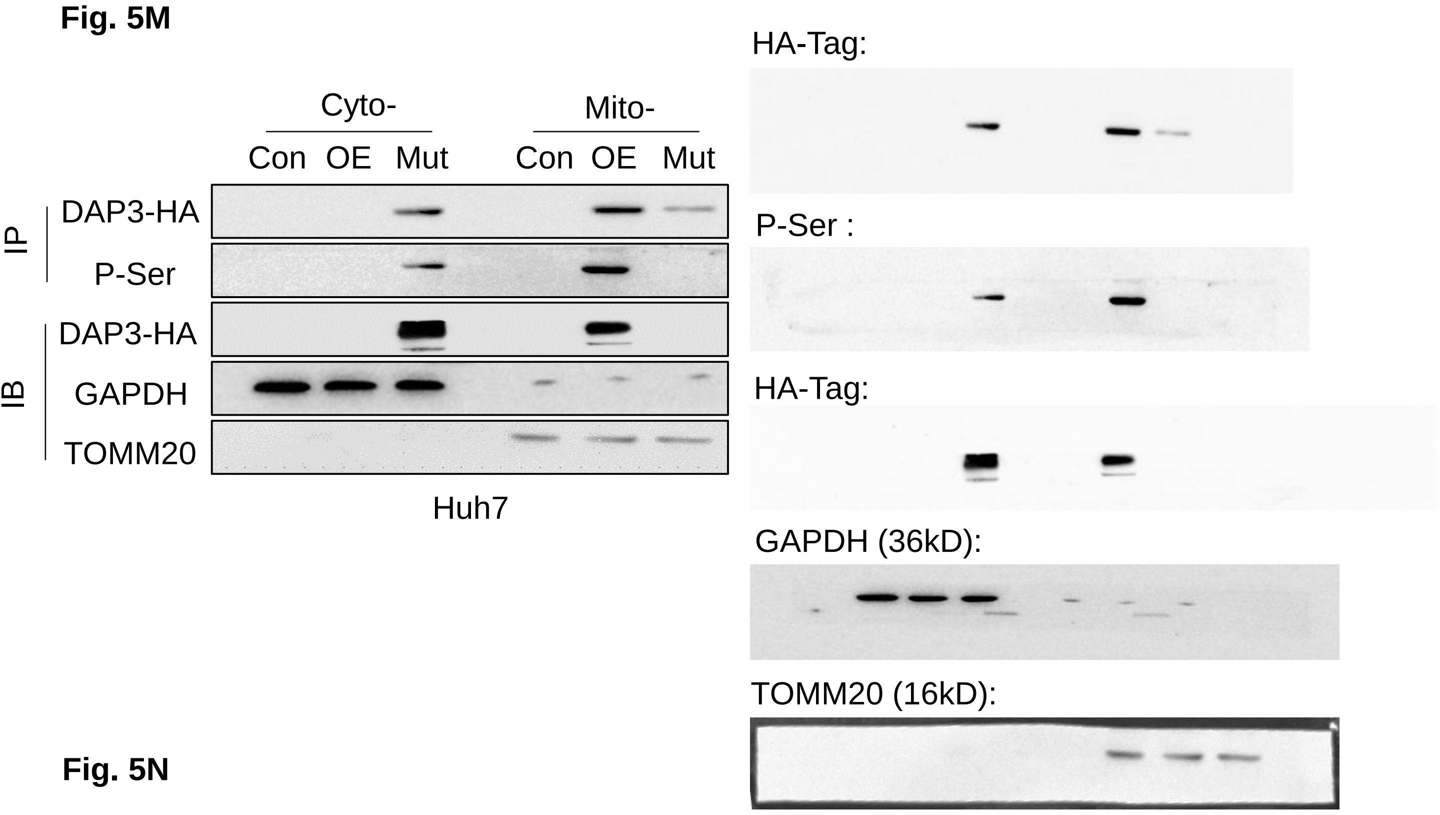

Fig. 5N

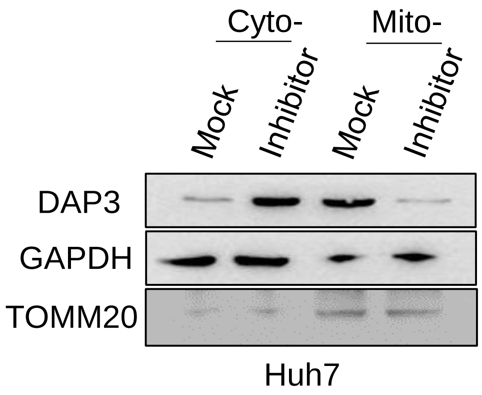

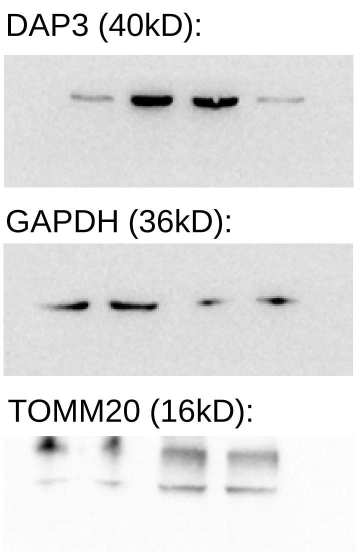

Fig. 5O

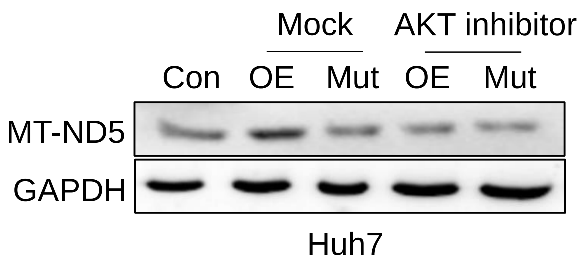

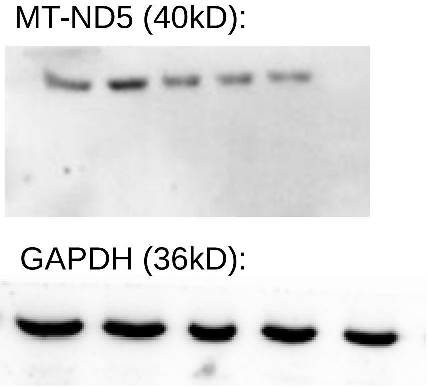

Fig. 6C

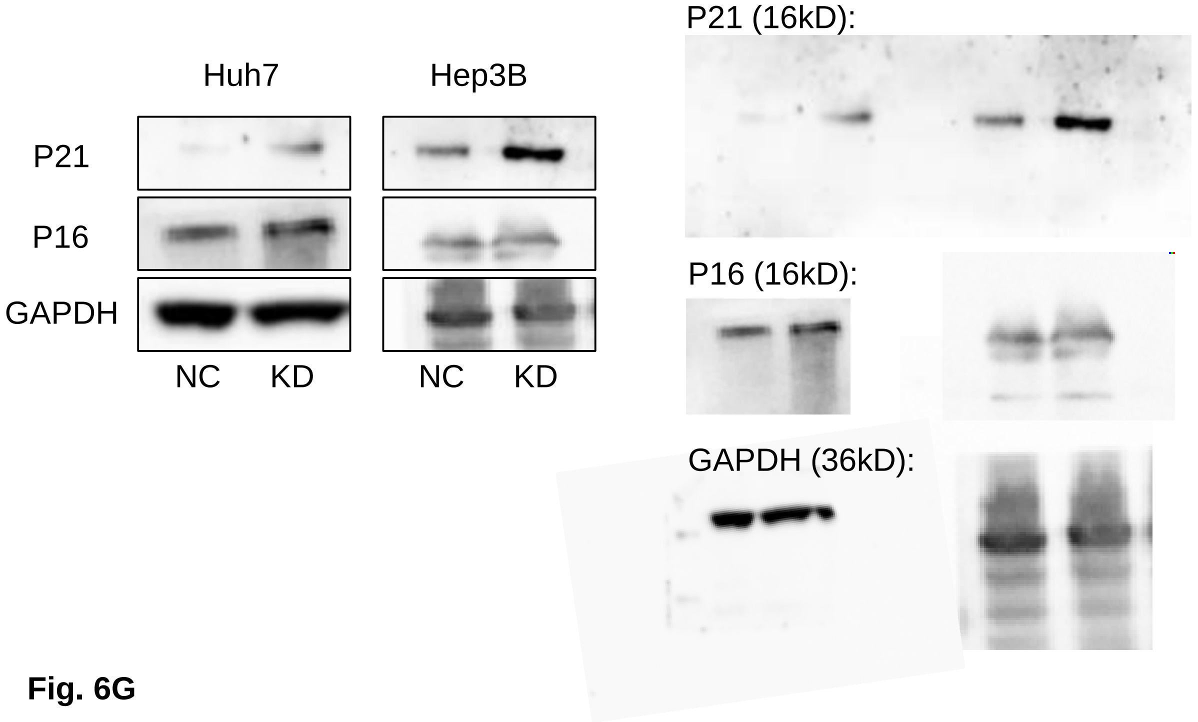

Fig. 6G

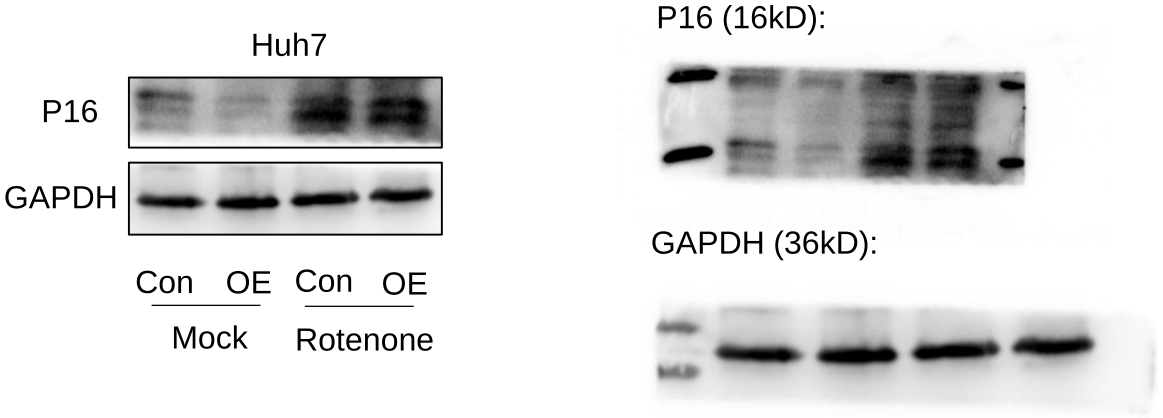

Fig. 6L

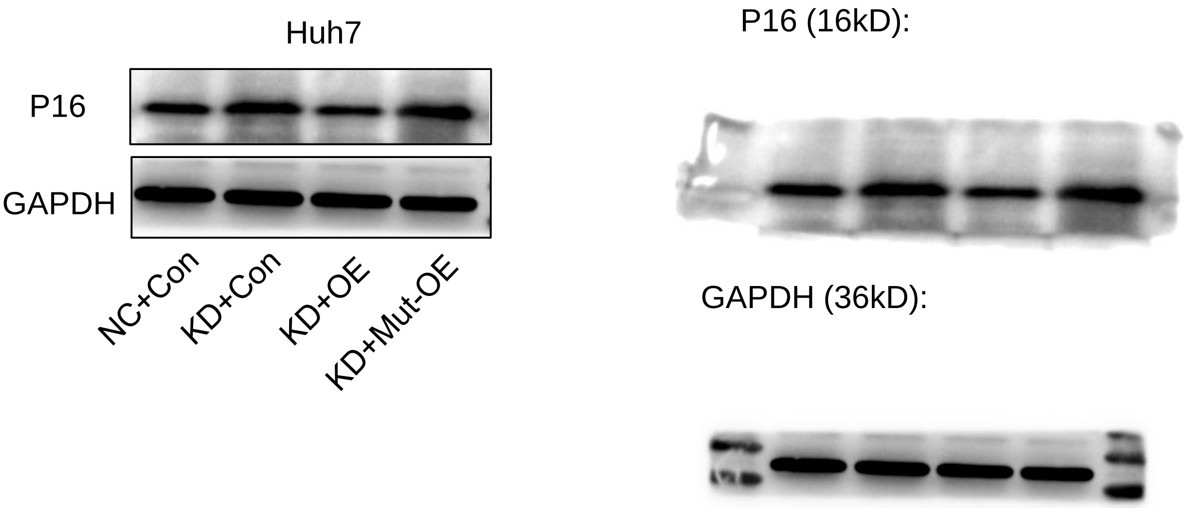

Fig. S2A

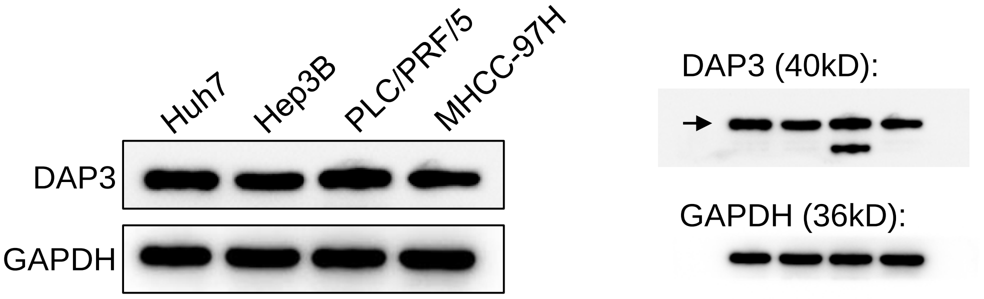

Fig. S2B

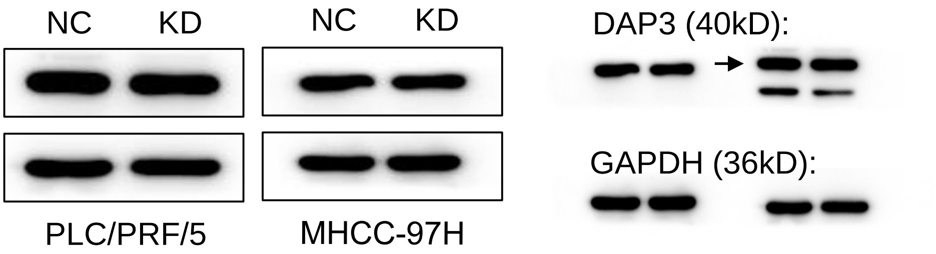

Fig. S2F

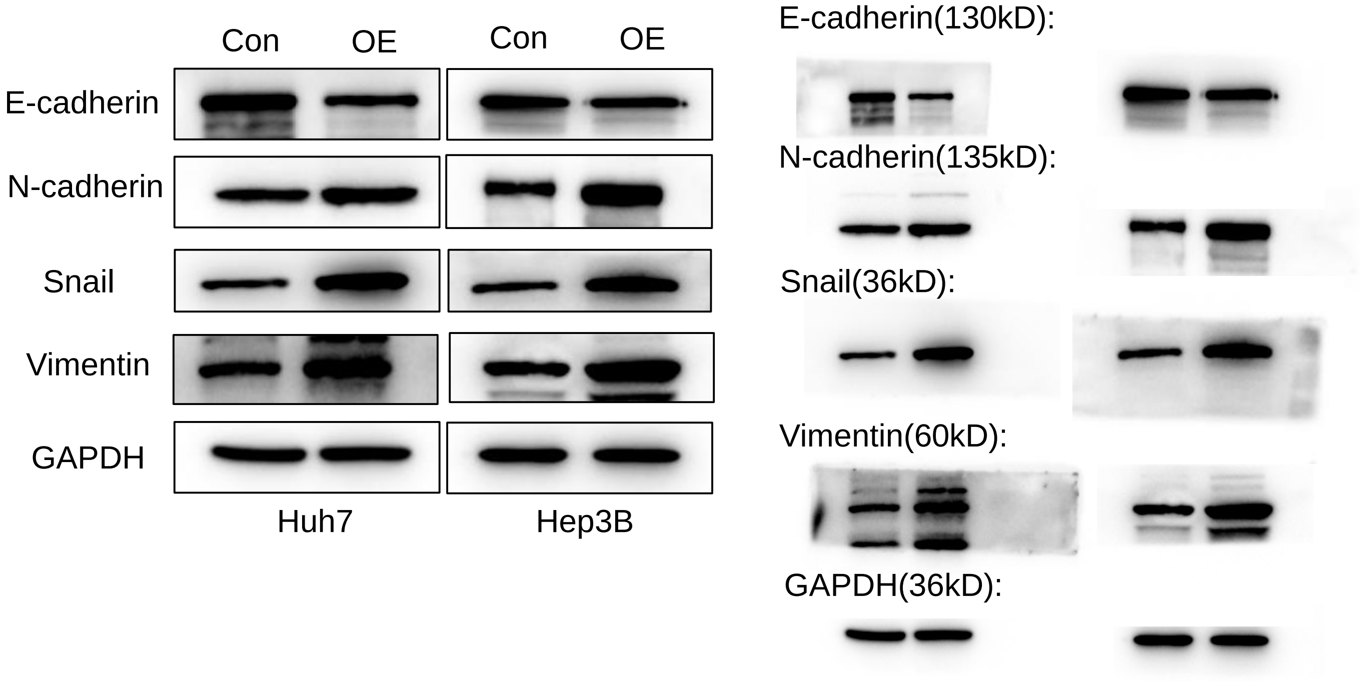

Fig. S4C

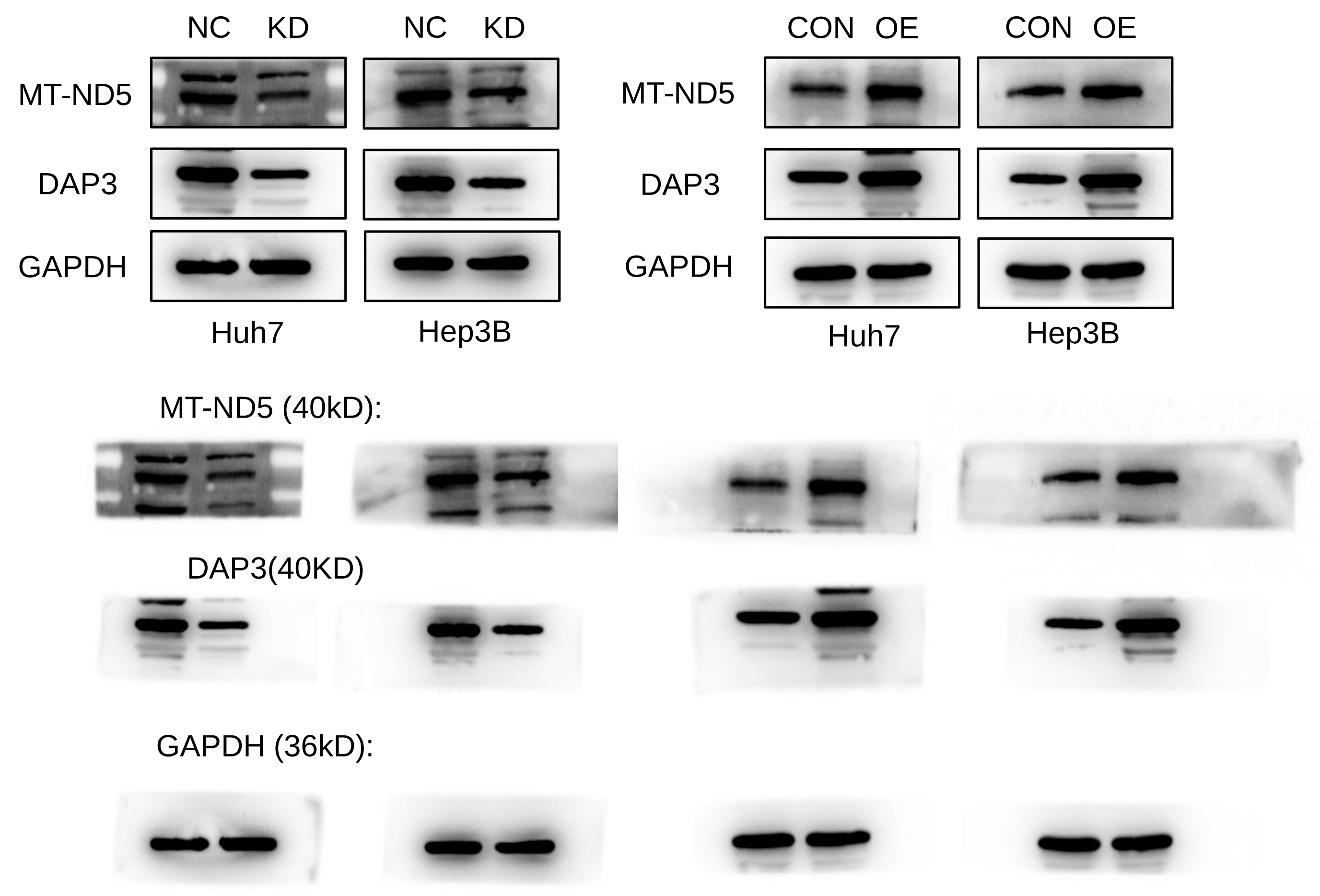

Fig. S4D

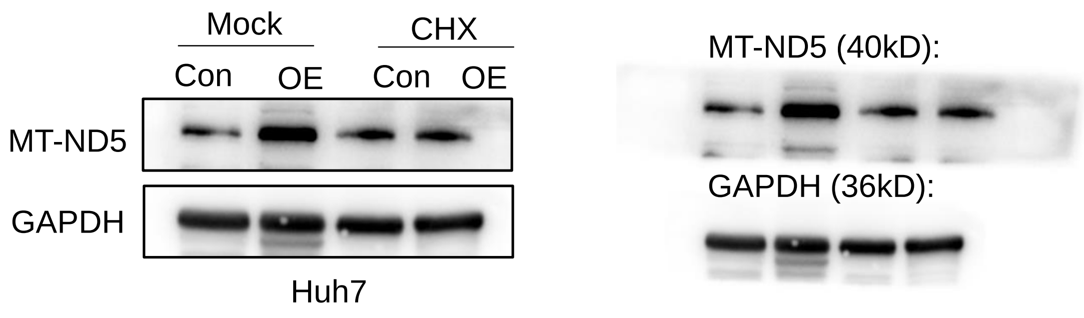

Fig. S4E

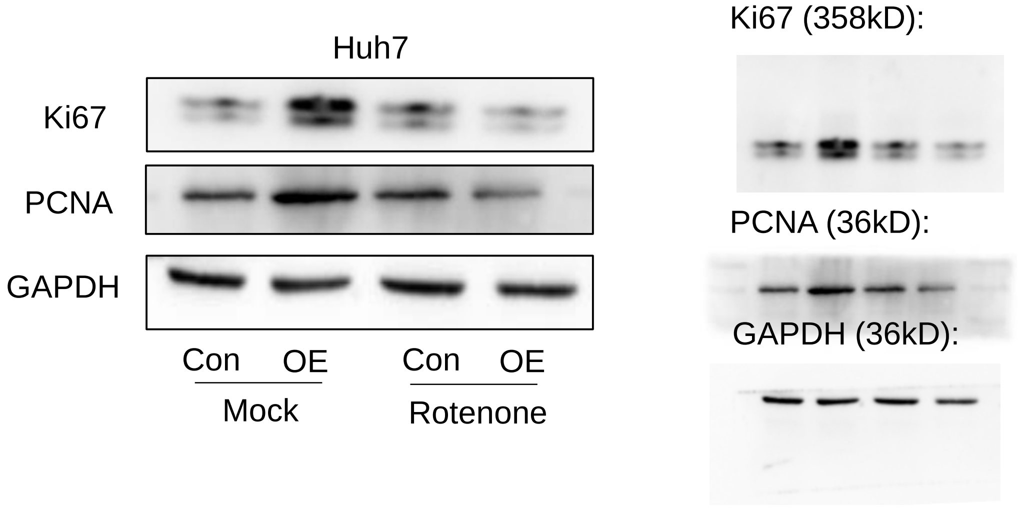

Supplement: Supplementary file 2 — Original Data [file 41419_2024_6912_MOESM2_ESM.pdf]
